# Perturbing chondroitin sulfate proteoglycan signaling through LAR and PTPσ receptors promotes a beneficial inflammatory response following spinal cord injury

**DOI:** 10.1186/s12974-018-1128-2

**Published:** 2018-03-20

**Authors:** Scott Dyck, Hardeep Kataria, Arsalan Alizadeh, Kallivalappil T. Santhosh, Bradley Lang, Jerry Silver, Soheila Karimi-Abdolrezaee

**Affiliations:** 1 Department of Physiology and Pathophysiology, the Regenerative Medicine Program, the Spinal Cord Research Center, University of Manitoba, 629-Basic Medical Sciences Building, 745 Bannatyne Avenue, Winnipeg, MB R3E 0J9 Canada; 2 Department of Neuroscience, Case Western Reserve University School of Medicine, Cleveland, OH 44106 USA

**Keywords:** Spinal cord injury, Chondroitin sulfate proteoglycans, Neuroinflammation, Microglia, Neural precursor cells, Leukocyte common antigen-related receptor, Protein tyrosine phosphatase sigma receptor

## Abstract

**Background:**

Traumatic spinal cord injury (SCI) results in upregulation of chondroitin sulfate proteoglycans (CSPGs) by reactive glia that impedes repair and regeneration in the spinal cord. Degradation of CSPGs is known to be beneficial in promoting endogenous repair mechanisms including axonal sprouting/regeneration, oligodendrocyte replacement, and remyelination, and is associated with improvements in functional outcomes after SCI. Recent evidence suggests that CSPGs may regulate secondary injury mechanisms by modulating neuroinflammation after SCI. To date, the role of CSPGs in SCI neuroinflammation remains largely unexplored. The recent discovery of CSPG-specific receptors, leukocyte common antigen-related (LAR) and protein tyrosine phosphatase-sigma (PTPσ), allows unraveling the cellular and molecular mechanisms of CSPGs in SCI. In the present study, we have employed parallel in vivo and in vitro approaches to dissect the role of CSPGs and their receptors LAR and PTPσ in modulating the inflammatory processes in the acute and subacute phases of SCI.

**Methods:**

In a clinically relevant model of compressive SCI in female Sprague Dawley rats, we targeted LAR and PTPσ by two intracellular functionally blocking peptides, termed ILP and ISP, respectively. We delivered ILP and ISP treatment intrathecally to the injured spinal cord in a sustainable manner by osmotic mini-pumps for various time-points post-SCI. We employed flow cytometry, Western blotting, and immunohistochemistry in rat SCI, as well as complementary in vitro studies in primary microglia cultures to address our questions.

**Results:**

We provide novel evidence that signifies a key immunomodulatory role for LAR and PTPσ receptors in SCI. We show that blocking LAR and PTPσ reduces the population of classically activated M1 microglia/macrophages, while promoting alternatively activated M2 microglia/macrophages and T regulatory cells. This shift was associated with a remarkable elevation in pro-regenerative immune mediators, interleukin-10 (IL-10), and Arginase-1. Our parallel in vitro studies in microglia identified that while CSPGs do not induce an M1 phenotype per se, they promote a pro-inflammatory phenotype. Interestingly, inhibiting LAR and PTPσ in M1 and M2 microglia positively modulates their inflammatory response in the presence of CSPGs, and harnesses their ability for phagocytosis and mobilization. Interestingly, our findings indicate that CSPGs regulate microglia, at least in part, through the activation of the Rho/ROCK pathway downstream of LAR and PTPσ.

**Conclusions:**

We have unveiled a novel role for LAR and PTPσ in regulating neuroinflammation in traumatic SCI. Our findings provide new insights into the mechanisms by which manipulation of CSPG signaling can promote recovery from SCI. More importantly, this work introduces the potential of ILP/ISP as a viable strategy for modulating the immune response following SCI and other neuroinflammatory conditions of the central nervous system.

**Electronic supplementary material:**

The online version of this article (10.1186/s12974-018-1128-2) contains supplementary material, which is available to authorized users.

## Background

Spinal cord injury (SCI) results in profound inhibitory modifications in the extracellular matrix (ECM), mainly driven by activated glia [1]. Dysregulation of the ECM contributes considerably to the formation of an impermissible microenvironment for repair and regeneration after SCI [2]. Dramatic upregulation of chondroitin sulfate proteoglycans (CSPGs) is considered a main inhibitory component of the post-SCI ECM [1]. CSPGs limit several endogenous repair mechanisms including axonal sprouting and regeneration as well as oligodendrocyte replacement and remyelination [3–8]. Different strategies have been employed to target CSPGs after SCI such as administration of chondroitinase ABC (ChABC) and xyloside treatments and genetic manipulations of *N*-acetylgalactosaminyl-transferase 1 and Sox9 [4, 6, 9–13]. Importantly, these studies have shown that inhibition of CSPGs improves recovery from SCI.

While the significance of CSPGs on spinal cord regeneration has been established, the cellular and molecular mechanisms of CSPGs in neuroinflammatory processes have yet to be elucidated. The recent discovery of specific CSPGs signaling receptors, leukocyte common antigen-related (LAR) and protein tyrosine phosphatase-sigma (PTPσ) [14, 15], provides the opportunity to uncover immunomodulatory mechanisms of CSPGs.

SCI triggers a complex immune response which is characterized by activation of resident microglia and recruitment of peripheral leucocytes to the site of injury. Currently, it is well accepted that neuroinflammation can be both beneficial and detrimental for SCI repair depending on the timing and phenotype of immune cells following injury [16–18]. Classically activated M1 microglia/macrophages are known to promote tissue damage through their production of pro-inflammatory cytokines (i.e., of IL-1β, TNFα, IL-6, IL-12, IL-23, and IFN-γ), proteases, and reactive oxygen species (ROS) [18]. Conversely, alternatively activated M2 microglia/macrophages are associated with phagocytosis of myelin debris and secretion of growth-promoting factors (i.e. IL-10, IGF-1) that support tissue repair [18–20]. After SCI in mice, there is initially a relatively equal number of M1 and M2 microglia/macrophage, which over time, shifts to an increasingly more prominent M1 inflammatory response [21]. This switch is deleterious to endogenous repair mechanisms as an M2 immune response has been shown to be essential for multiple repair processes including axonal sprouting and regeneration in SCI [19, 20, 22, 23] and oligodendrocyte maturation and remyelination in multiple sclerosis (MS) [24]. The microenvironment of SCI appears to favor an M1 phenotype as transplantation of M2 macrophages into the injured spinal cord at 7 days post-SCI drives the majority of these cells to adopt an M1 phenotype 3 days post-transplantation [21, 25, 26]. Thus, a better understanding of the endogenous mechanisms that regulate immune cells in the injured spinal cord will allow for the development of immunomodulatory therapies for SCI.

Evidence suggests that targeting the upregulated levels of CSPGs by ChABC treatment can promote an M2 inflammatory response after SCI [27, 28]. Here, we investigated whether CSPGs modulate inflammatory processes after SCI through the activation of LAR and PTPσ. Studies by our group and others have identified a critical role for LAR and PTPσ in mediating CSPG effects on multiple cell types including neural precursor cells (NPCs) [3], oligodendrocyte progenitor cells (OPCs) [29], and neurons [14, 15, 30, 31], and genetic manipulation of their expression is sufficient to limit the effects of CSPGs in vitro. Membrane-permeable intracellular LAR peptide (ILP) and intracellular sigma peptide (ISP) have been developed to inhibit LAR and PTPσ receptors [30, 32]. These peptides were designed to bind to a highly conserved 24-amino acid intracellular wedge domain and thereby modulate the catalytic activity of these receptors. Efficacy of ILP and ISP in blocking CSPG effects has been demonstrated in vitro and in SCI [15, 30, 32]. In rat SCI, our group and others have demonstrated that pharmacological inhibition of LAR and PTPσ by ISP and ILP remarkably increases sprouting of serotonergic fibers associated with improved functional recovery [15, 30].

In the present study, by utilizing ILP and ISP in a clinically relevant model of compressive/contusive SCI in the rat, we have unveiled a novel immunomodulatory role for CSPGs that is mediated through LAR and PTPσ receptors. We show that blocking LAR and PTPσ with ILP and ISP fosters an increase in the number of M2 microglia/macrophages and T regulatory cells after SCI that is marked by elevated levels of interleukin-10 (IL-10). Our parallel in vitro studies on microglia uncovered that while CSPGs themselves do not induce an M1 phenotype, their presence in the milieu of M1 microglia promotes their pro-inflammatory phenotype. Importantly, inhibiting LAR and PTPσ in M1 microglia attenuated their Interleukin 1 beta (IL-1β) expression in the presence of CSPGs and prompted their phagocytic ability and mobilization. Moreover, we have identified that CSPGs regulate microglia, at least partially, by activation of the Rho/ROCK pathway in which can be attenuated by the inhibition of LAR and PTPσ. Taken together, our findings provide novel insights into the cellular and molecular mechanisms by which modulation of CSPGs or their receptors, LAR and PTPσ, can improve endogenous repair mechanisms and neurological recovery following SCI [4–6, 15, 30, 33]. We also provide the first evidence suggesting the potential of ILP and ISP as a candidate immunotherapy for SCI.

## Methods

### Animals and animal care

All experimental protocols in this study were approved by the Animals Care Committee of the University of Manitoba in accordance with the guidelines and policies established by the Canadian Council of Animal Care (CCAC). For in vivo studies, a total of 112 adult female Sprague Dawley (SD) rats (250 g), and for in vitro experiments, 6 C75BL/6 mice (8 weeks) and 44 postnatal (P1–P3) SD pups were used (provided by the Central Animal Care Facility at the University of Manitoba, Canada). Adult female rats were housed in standard plastic cages at 22 °C before SCI and at 26 °C afterwards in a 12:12 h light/dark cycle. Pelleted food and drinking water were available ad libitum. Hardwood sawdust bedding was used before SCI surgeries and replaced by soft paper bedding after SCI to prevent skin erosions and urine scalding.

### Rat model of compressive spinal cord injury

We employed a clinically relevant clip-compression model of SCI that has been extensively characterized and employed for SCI pathophysiology and therapeutics by our group and others [5, 6, 34–36]. Under sterile conditions, general anesthesia was induced by inhalation of a mixture of O_2_ (2%) and Isoflurane (4%) via a mask integrated into a surgical stereotaxic frame. After deep anesthesia was achieved, for maintenance, isoflurane was reduced to 2%. The surgical area was shaved and disinfected with 70% ethanol and Povidone iodine. A midline incision was made at the thoracic area (T4–T9) and skin and superficial muscles were retracted. The rats received a T6–T8 laminectomy and then, a 35 g aneurysm clip (University Health Network, Toronto, Ontario, Canada) was applied extradurally for 1 min at the level of T7 of the spinal cord to induce a compression injury. For surgical pain and discomfort management, each animal received one single injection of meloxicam (Metacam^®^ Boehringer Ingelheim GmbH, 2 mg/kg) prior to the surgery followed by four doses of buprenorphine (Vetergesic, 0.03 mg/kg), the first given immediately after SCI and the following three doses at 8-h intervals. Animals also received 5 ml of 0.9% saline subcutaneously after SCI and thereafter as needed to prevent dehydration. Additionally, animals received oral Clavamox^®^ (Amoxicillin plus Clavulanic Acid, Pfizer) in their drinking water starting 2 days before surgeries until 3 days post-operation to prevent trauma-induced hematuria and bladder infection. SCI rats were examined daily to monitor their recovery and their bladders were expressed manually three times a day until the return of reflexive bladder control.

### Experimental groups and treatments

**Fig. 1:**
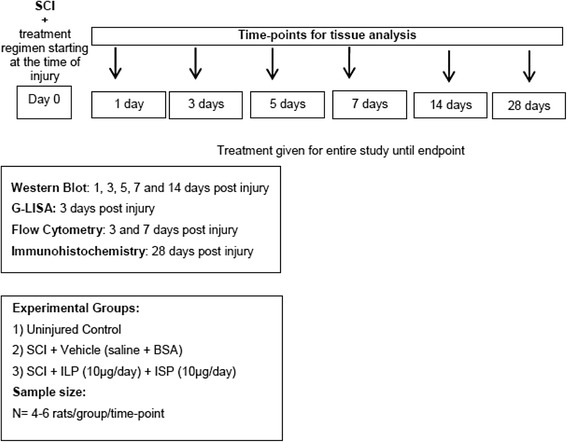
Summary of experimental procedures, treatment groups, and time-points for in vivo experiments

Prior to the SCI procedure, animals were randomly assigned to three experimental groups: (1) uninjured; (2) SCI/vehicle control, receiving vehicle solution used for preparation of ILP + ISP (0.1% bovine serum albumin, BSA in 0.9% saline); and (3) SCI/ ILP (NH2-GRKKRRQRRRCDLADNIERLKANDGLKFSQEYESI-NH2, CS Bio Co.) + ISP (NH2-GRKKRRQRRRCDMAEHMERLKANDSLKLSQEYESI-NH2, CS Bio Co.) (10 μg each/day) (Fig. 1). The dose of ILP and ISP was previously determined in our previous SCI studies [30]. We conducted time-point analyses at 1, 3, 5, 7, and 14 days. For all time-points (Fig. 1), treatment was administered intrathecally at the time of SCI using a mini-osmotic pump and an indwelling intrathecal catheter inserted into the subarachnoid space surrounding the lesion site with the tip of the catheter located rostral to the lesion (Alzet—model 2001D, 1003D, 2001, 2002, and 2004). Based on the delivery rate of each pump, ILP and ISP concentration was adjusted to ensure sustained delivery of comparable dose per day for each time-point. The SCI/vehicle control group also received vehicle via osmotic pumps in the same manner as the treatment groups. Uninjured animals did not undergo any surgical procedure in this study.

### Tissue processing

Tissue harvesting was performed at the end of the treatment period. Animals that received treatment at the time of injury were euthanized at 1, 3, 5, 7, 14, and 28 days post-SCI (*N* = 4–6 animals/group/time-point). SCI rats were deeply anesthetized with a mixture of 40% isoflurane/60% propylene glycol (Fisher Scientific, Pittsburgh, PA, USA). For molecular analyses, rats were perfused transcardially with 0.1 M phosphate-buffered saline (PBS) to remove blood. Once blood was removed, freshly dissected spinal cords were placed in ice-cold artificial cerebrospinal fluid (aCSF), and cleaned of meninges and nerve roots. Five millimeters of spinal cord tissue centered at the injury site were dissected and processed either for Western blotting or slot blotting, as described subsequently. For histological analyses, rats under deep anesthesia were perfused transcardially with 2.5% paraformaldehyde (PFA) in 0.1 M PBS, pH 7.4. The spinal cords were excised and subsequently postfixed in the perfusing solution plus 10% sucrose overnight at 4 °C. Then, the tissues were further cryoprotected in 20% sucrose in PBS for 24–48 h at 4 °C. A 1.5–2 cm length of the spinal cord tissue centered at the injury site was dissected and immersed in tissue-embedding medium (Tissue-Tek™ CRYO-O.C.T Compound, Electron Microscopy Sciences) on dry ice. Cross sections (35 μm) were cut serially on a cryostat (Leica) and mounted onto Superfrost® Plus Micro Slides (Fisher Scientific) and stored at − 80 °C until the immunostaining procedure was undertaken.

### Immunohistochemistry on tissue sections

Frozen spinal cord cross-sections were air-dried at room temperature for 30 min. The injury epicenter for each sample was determined by Hematoxylin and Eosin (H/E) staining and the section near the midpoint of the lesion with the largest injury area was considered as the epicenter. Slides were permeabilized with PBS for 5 min and then blocked for 1 h at room temperature using 5% skim milk, 1% BSA, 0.05% Triton-X in 0.1 M PBS. This blocking solution was used for all immunostaining procedures unless mentioned otherwise. Tissue sections were then incubated overnight at 4 °C with primary antibodies (Table 1) diluted in the blocking solution. Sections were washed three times in PBS then incubated with Alexa 568 goat anti-mouse, rabbit, or goat secondary antibody (1:400; Invitrogen) for 1.5 h. In a double-staining procedure, the tissue sections were treated with a second primary antibody then incubated with Alexa 488 goat anti-mouse or rabbit secondary antibody (1:400; Invitrogen). The slides were washed three times with PBS and incubated with DAPI as a nuclear counterstain.

**Table 1:** List of antibodies used in this study

Antibody	Source	Usage	Dilution factor
Actin	Sigma (Rabbit, A2066)	WB	1:300
Actin	Chemicon (Mouse, MAB1501R)	WB	1:300
Arginase-1 (5% BSA blocking)	Cell Signaling (Rabbit, 9819S)	WB	1:1000
BrdU	Santa Cruz (Mouse, 555,627)	ICC	1:500
CD3 (1% BSA, 5% goat serum blocking)	Abcam (Rabbit, AB5690)	IHC	1:200
CD4	Cedarlane (Sheep, AF6439)	WB	1:100
CD86	Abcam (Rabbit, AB53004)	ICC	1:60
CS56	Sigma (Mouse, C8035)	IHC	1:150
DAPI	Sigma (D9542)	ICC IHC	1:10000
FOXP3	Cedarlane (Rat, 14-5773-82)	WB IHC	1:200 1:100
Iba1	Wako (Rabbit, 016-20,001)	WB	1:1000
Iba1	Wako (Rabbit, 019-19,741)	ICC IHC	1:500 1:500
IL-10	Cedarlane (Mouse, MAB519)	WB	1:250
IL-10 (1% BSA, 5% Goat serum blocking)	R&D Systems (Rat, MAB417)	IHC	1:200
IL-1β	Serotec (Rabbit, AAR15G)	WB	1:1000
GFAP	Chemicon (Mouse, MAB360)	ICC	1:800
GFAP	Cell Signaling (Mouse, #3670S)	WB	1:5000
GFAP	Chicken	IHC	1:800
GAPDH	Santa Cruz (Rabbit, sc-25,778)	WB	1:1000
LAR	Santa Cruz (Rabbit, sc-25,434)	ICC	1:50
LAR	BD (Mouse 610,351)	WB	1:250
Mannose receptor	Abcam (Rabbit, ab64693)	ICC	1:400
Olig2	Chemicon (Rabbit, AB9610)	ICC	1:2000
OX42 (CD11b)	Serotec (Mouse, MCA275G)	IHC	1:100
PTPσ	R&D (Goat, AF3430)	ICC	1:50
TNFα	Serotec (Rabbit, AAR33)	WB	1:1000

*ICC* immunocytochemistry, *IHC* immunohistochemistry, *WB* Western blot

### Assessment of GFAP and CS56 Immunointensity in SCI

CSPG and glial fibrillary acidic protein (GFAP) immunostaining was imaged using a Zeiss AxioImager M2 fluorescence microscope (Zeiss) (*n* = 4–6 animals/group/time-point). We imaged the entire cross-section of the spinal cord with a × 10 objective using Zen tiling software (Zeiss). Imaging procedures were conducted under the same condition and consistent exposure time for all spinal cord sections as we described previously [6, 36, 37]. Using NIH ImageJ software (imagej.nih.gov), we traced the cross-sectional area of the spinal cord and measured the immunodensity of GFAP and CSPGs in spinal cord cross-sections representing the injury epicenter as well as 1 mm rostral and caudal to the lesion center. After setting the threshold automatically, immunodensity above threshold was calculated. To account for variation in the size of spinal cord cross sections, the following formula was used to calculate the percentage of CSPG and GFAP area: normalized immunodensity of tissue section *X* = total immunodensity of tissue section *X* / total area of the spinal cord section *X*.

### Western blotting

For Western blotting, spinal cord tissue or cultured cells were homogenized in RIPA buffer (Thermo Fisher) containing SigmaFast Protease Inhibitor (Sigma). A total of 10 to 50 μg of proteins were then loaded onto gel and transferred to a nitrocellulose membrane (Bio-Rad). The membranes were then blocked with 5% non-fat milk in Tween Tris-buffered saline (TTBS) and incubated overnight at 4 °C with different antibodies (Table 1) diluted in the blocking solution. The membranes were washed and incubated with HRP-conjugated goat anti-mouse, anti-sheep, anti-rat, or anti-rabbit antibodies (1:4000, Bio-Rad). Membranes were then incubated in ECL plus immunoblotting detection reagents (Thermo Scientific) according to the manufacturer's specifications. For arginase-1 antibody, the blocking solution was made of 3% BSA in TTBS. Immunoreactive bands were quantified using AlphaEaseFC (FluorChem, 8900). To control for equal protein loading, membranes were re-probed for actin antibody.

### Myeloperoxidase assay in SCI

Myeloperoxidase (MPO) enzyme activity was assessed as previously described [38]. Briefly, MPO assay buffer was prepared by dissolving 80 mmol phosphate buffer (pH 5.4), 0.5% hexadecyltrimethyl ammonium bromide, and 1.6 mmol tetramethylbenzidine in dimethylformamide and 2 mmol H_2_O_2_. Then, 200 μl of MPO assay buffer was added to 50 μg of tissue lysate at 37 °C. Change in absorbance per minute was assessed at 655 nm. MPO activity was expressed as the amount of the enzyme producing one absorbance change per minute.

### Gelatin Zymography on SCI tissues

To assess matrix metalloproteinases (MMP)-2 and MMP-9 enzymatic activity in the injured spinal cord tissue, 50 μg of protein obtained from the SCI tissue was loaded on 10% SDS-polyacrylamide gels, co-polymerized with 1 mg/ml gelatin as a substrate and were separated by electrophoresis. Proteins were renatured by 2.5% Triton X-100 to restore gelatinase activity. Gels were then incubated with developing buffer for 48–72 h at 37 °C to allow gelatinase activity of MMP-2 and MMP-9. Gels were stained with Coomassie blue for 30 min and de-stained in 30% ethanol/10% acetic acid until appropriate color contrast was achieved. Areas of gelatinase activity appeared as clear bands against a dark blue background. MMPs were identified based on their molecular weight and their density was measured as described in our Western blot procedures.

### Flow cytometric assessment in rat SCI

To study changes in immune cell populations and phenotypes in the injured spinal cord, we performed flow cytometry on spinal cord tissue [39]. At 3 and 7 days post-injury, (*N* = 5/experimental group), animals were anesthetized and euthanized. The vertebral columns were excised and placed on dry ice for 5 min. The spinal cords were exposed using a laminectomy and 1.5 cm of tissue centered at the injury epicenter was excised, minced, and enzymatically dissociated by incubating with 2.5 mg trypsin + 5 mg collagenase in 5 ml DMEM media for 20 min at 37 °C. Cells were pelleted and reconstituted in 6 ml of HBSS and overlaid on OptiPrep® (Sigma-Aldrich, D1556) gradient for separation of myelin debris and incubated with red blood cell (RBC) lysis buffer (Biolegend, 420301) prior to counting. An average of 7.5 million cells was harvested from each animal. For each antibody panel, 2 million cells per animal were used. Non-specific binding sites were blocked using 10% normal mouse serum for 30 min (Invitrogen, 10,410). Cells were then incubated in an antibody cocktail containing surface antibodies for each panel for 30 min away from light at 4 °C. Next, cells were washed and fixed using BD™ Cytofix Fixation buffer for 15 min at 4 °C (BD, 554655). To stain for intracellular markers, cells were incubated with permeabilizing buffer (0.1% saponin + 10% FBS in HBSS) for 30 min then incubated with a cocktail of intracellular antibodies for 30 min in the dark. After washing, cells were reconstituted with 500 μl of flow cytometry staining buffer and analyzed using BD FACS Canto II flow cytometer counting 200,000 events per sample. Compensation was done prior to acquisition using single-stained beads (OneComp eBeads, 501129031, eBioscience). For each antibody panel, proper isotype controls were used to account for non-specific antibody binding (Additional file 1: Figure S1 and Additional file 2: Figure S2). Our flow gating strategy is depicted in Additional file 3: Figure S3 and Additional file 4: Figure S4. Additionally, flow cytometry antibodies and their isotype controls are listed in Table 2.

**Table 2:** List of flow cytometry antibodies used in this study

Antibody	Color	Company-Cat number	Dilution factor
CD3	PerCP	eBioscience, 46-00-30-82	1:20
CD4	BV510	BD, 740138	1:20
Ms IgG2a, k	BV510	BD, 563027	1:20
CD45	APC-Cy7	BD, 561586	1:20
Ms IgG1, k	APC-Cy7	BD, 557873	1:20
IFN-γ	FITC	BD, 559498	1:20
Ms IgG1, k	FITC	BD, 554679	1:20
IL-10	PE	BD, 555088	1:20
Ms IgG2b, k	PE	BD, 555058	1:20
FoxP3	APC	eBioscience, 17-5773-80	1:20
Ms IgG2a, k	APC	eBioscience, 17-4724-42	1:20
CD68	FITC	Bio-Rad, MCA341F	1:20
Ms IgG1	FITC	Bio-Rad, MCA1209F	1:20
CD163	PE	Bio-Rad, MCA342R	1:20
Ms IgG1	PE	Bio-Rad, MCA1209PE	1:20
CD86	BV-421	BD, 743211	1:20
Ms IgG1, k	BV-421	BD, 562438	1:20
IL-10	Alexa 647	BD, 562156	1:20
Ms IgG2b, k	Alexa 647	BD, 557903	1:20
TNFα	PE-Cy7	eBiosicence, 25-7423	1:20
Armenian Hamster IgG isotype control	PE-Cy7	eBioscience, 25-4888-82	1:20

### Culture and isolation of primary microglia

Primary microglia were isolated from mixed glial cultures as described previously [40]. Briefly, postnatal (P1–P3) rat pup cortices were mechanically dissociated and the cells passed first through a 70 μm and then a 45 μm cell strainer. The cell solution was then plated into 75 cm^2^ flasks in DMEM/F12 (1:1) supplemented with 10% fetal bovine serum and 1% penicillin/streptomycin/neomycin (PSN). Mixed glia cultures were maintained with media change every 3 days until confluency (2–3 weeks). Mixed glia cultures were then shaken at 200 rpm for 6 h at 37 °C to separate microglia from underlying astrocytes. The microglia were seeded over poly-d-lysine (PDL, 0.1 mg/ml, Sigma) coated dishes in 50% fresh media (DMEM/F12 plus 10% FBS) and 50% mixed glia conditioned media (collected and filter sterilized after shaking). Purity of microglia in these cultures was over 90% (data not shown).

### Microglia polarization

Primary microglia were plated at a density of 500,000 cells per well of a 6-well plate in 50% glia conditioned media and 50% DMEM/F12 plus 10% FBS media. Once the majority of microglia were attached and began to spread their cell processes (after 1–2 days), their media was changed and microglia were polarized to M0 (untreated), M1 (co-treatment of tumor necrosis factor-α (TNF-α, 40 ng/ml) and interferon gamma (IFNγ, 50 ng/ml)), and M2 (interleukin-10 (IL-10, 10 ng/ml)). Two days following cell activation, microglia-conditioned media was harvested and stored at − 80 ºC for future experiments. Microglia polarization was confirmed using ELISA, the Griess assay, and immunocytochemical analyses for various M1 and M2 markers.

### Plating microglia on CSPG substrates

All tissue-culture dishes were first coated with PDL (0.1 mg/ml, Sigma) overnight at room temperature, followed by a mixture of CSPGs (5 μg/ml, Millipore, cc117) for 6 h at 37 °C as we described previously [3]. Of note, CSPGs used in this study contained a mixture of neurocan, phosphacan, versican, and aggrecan. Where appropriate, chondroitinase ABC (ChABC, 0.1 U/ml Sigma, C3667-10UN) was added with the CSPG mixture during the coating step. M0, M1, and M2 microglia cultures were activated 3 h prior to cell plating (see Table 3). Three hours following cell activation, microglia were lifted and plated onto various substrates including (1) PDL, (2) PDL + CSPGs (5 μg/ml), and (3) PDL + CSPGs (5 μg/ml) + ChABC. Two days following treatments, microglia were assessed for various outcomes including cytokine release, nitric oxide activity, and phagocytosis.

**Table 3:** Assessment of CSPG effects on microglia experimental groups

Experimental group	Treatment	Cell plating (3 h post treatment)
M0 (no treatment)	–	1) PDL 2) PDL + CSPGs (5 μg/ml) 3) PDL + CSPGs (5 μg/ml) + ChABC (0.1 U/ml)
	ILP (10 μM) + ISP (10 μM)	1) PDL 2) PDL + CSPGs (5 μg/ml)
	Y-27632 (ROCK inhibitor, 10 μM)	1) PDL 2) PDL + CSPGs (5 μg/ml)
	TAT (20 μM)	1) PDL 2) PDL + CSPGs (5 μg/ml)
	IMP (10 μM)	1) PDL 2) PDL + CSPGs (5 μg/ml)
M1 (40 ng/ml TNFα + 50 ng/ml IFNγ)	–	1) PDL 2) PDL + CSPGs (5 μg/ml) 3) PDL + CSPGs (5 μg/ml) + ChABC (0.1 U/ml)
	ILP (10 μM) + ISP (10 μM)	1) PDL 2) PDL + CSPGs (5 μg/ml)
	Y-27632 (ROCK inhibitor, 10 μM)	1) PDL 2) PDL + CSPGs (5 μg/ml)
	TAT (20 μM)	1) PDL 2) PDL + CSPGs (5 μg/ml)
	IMP (10 μM)	1) PDL 2) PDL + CSPGs (5 μg/ml)
M2 (10 ng/ml IL10)	–	1) PDL 2) PDL + CSPGs (5 μg/ml) 3) PDL + CSPGs (5 μg/ml) + ChABC (0.1 U/ml)
	ILP (10 μM) + ISP (10 μM)	1) PDL 2) PDL + CSPGs (5 μg/ml)
	Y-27632 (ROCK inhibitor, 10 μM)	1) PDL 2) PDL + CSPGs (5 μg/ml)
	TAT (20 μM)	1) PDL 2) PDL + CSPGs (5 μg/ml)
	IMP (10 μM)	1) PDL 2) PDL + CSPGs (5 μg/ml)

### In vitro assessment of ILP and ISP peptides in blocking CSPG effects on microglia

ILP and ISP peptides, against LAR and PTPσ, respectively, were used (10 μM) as we described previously [30]. A control peptide intracellular Mu peptide (IMP, NH2-LLQHITQMKCAEGYGFKEEYESGRKKRRQRRRC-NH2, CS Bio Co.) was also used to assess specificity of ILP and ISP effects [32]. All peptides contained a transactivator of transcription of human immunodeficiency (TAT), domain (GRKKRRQRRRC) to facilitate intracellular delivery. Additionally, our previous work in NPCs revealed that CSPG signaling is mediated through the Rho/ROCK pathway [3]. Therefore, we also treated microglial cultures with Y-27632 (10 μM), a ROCK inhibitor. Microglia were dissociated and plated onto tissue culture surfaces containing one of two conditions: (1) PDL; (2) PDL + CSPGs. For both experimental conditions, microglia were pretreated with IMP (control), TAT peptide (control), Y-27632, ILP, ISP, or ILP and ISP for 30 min.

### Microglial phagocytosis assay

For the phagocytosis assay, green fluorescent latex beads of 1 μm diameter (Sigma, L1030) were preopsinized by adding 1 μl of the beads to 5 μl of FBS for 1 h at 37 °C [41]. Fluorescent beads were then diluted in microglia media and added to microglia cultures at a final concentration of 0.01% (*v*/*v*). After 1 h, microglia media was removed and cells were washed twice gently with PBS then fixed in 3% PFA for 15 min. Microglia were stained with DAPI for 15 min and imaged. The percentage of DAPI+ cells containing fluorescent beads was quantified. Engulfment of fluorescent beads was confirmed by Z-stack imaging and co-localization of Iba1+ cells with green fluorescent signal.

### Griess nitrite assay

The Griess assay (Promega, Fisher) was used to measure nitrite levels as a representative of NO activity in microglia-conditioned media (MCM) collected at 48 h after microglial activation according to the manufacturer's instructions [42, 43]. To eliminate any possible interference in Griess assay readings, phenol red free media was used for these experiments.

### Microglia migration assay

Microglia were plated onto PDL-coated or PDL + CSPGs (5 μg/ml) poly-carbonate transwell culture inserts (Corning, 100,000 cells per transwell) in serum-free media (SFM) in a 24-well plate. Microglia SFM containing C5a (R&D Systems, CL7336R 30 nM) was added to the bottom chamber to act as a chemoattractant [44]. Cells were allowed to migrate for 16 h at 37 °C and were fixed for 30 min with 3% PFA and stained for DAPI (1:5000). Non-migrated cells on the upper side of the transwell were gently scraped off with a cotton swab and the migrated cells were visualized by DAPI staining as described previously [24]. Eight images were taken at × 40 magnification to determine the total number of DAPI+ migrated cells.

### ElISA

We used commercial ELISA kits for cytokine analysis in vitro (DuoSet ELISA Development System; R&D Systems; #DY522 for IL-10; #DY501 for IL-1β) to specifically detect IL-10 and IL-1β in microglia-conditioned media. The assay was performed according to the manufacturer’s instructions, with standards (62.5–4000 pg/ml for both assays) and loading of 50 μl of microglia-conditioned media per sample.

### G-LISA

GTP-bound RhoA was assessed with a G-LISA assay (Cytoskeleton, Inc. Denver, CO, USA). For in vivo tissue samples, spinal cord tissue was excised from the spinal cord at 3 days post injury and 1 cm of spinal cord tissue centered at the injury epicenter was flash frozen in liquid nitrogen. For in vitro Rho activity assessment, tumor necrosis factor-α (TNFα) (40 ng/ml) + IFNγ (50 ng/ml) treated M1 microglia were grown on PDL or PDL + CSPGs with and without ILP (10 μM) and ISP (10 μM) for 1 day then harvested on ice and flash frozen in liquid nitrogen. For G-LISA assessment, 50 μg of protein was used for both in vivo and in vitro assessments. G-LISA was performed as per manufacturer’s instructions.

### Isolation and culturing of spinal cord adult neural precursor cells

Adult NPCs were isolated from the spinal cord of C57BL/6 mouse (8 weeks of age) as we described previously [36]. Briefly, mice were deeply anesthetized by placing them in a bell jar saturated with a mixture of 40% isoflurane/60% propylene glycol. Deep anesthesia was confirmed by lack of pedal reflexes. Mice were then killed by decapitation, and their spinal cords were excised under sterile conditions and transferred to an artificial cerebrospinal fluid (aCSF) solution (containing 124 mM NaCl, 3 mM KCl, 1 mM NaHPO4, 26 mM NaHCO3, 1.5 mM MgSO4, 1.5 mM CaCl2, and 10 mM glucose). Spinal cords were cleaned of meninges and nerve roots, and were then subjected to a papain enzymatic solution (Worthington Biochemical Corporation) for 50 min at 37 °C. The solution was then replaced by a papain inhibitor mixture and cells were passed through a 70 μm cell strainer. Cellular components were isolated through 7.5% BSA gradient and resuspended in serum-free medium (SFM, 100 ml) containing 84 ml of DMEM/F12 (Invitrogen), 2 ml of 30% glucose, 1.5 ml of 7.5% NaHCO_3_, 0.5 ml of 1 M HEPES, 10 mg of transferrin, 2.5 mg of insulin, 0.96 mg of putrescine, 1 μl of selenium, 1 μl of progesterone, 1% l-glutamine, 1% penicillin/streptomycin/neomycin (PSN), and growth factors: 1 μg of fibroblast growth factor-2 (FGF2, Fisher, CB40060A), 2 μg of epidermal growth factor (EGF, Sigma, E-4127), and 200 μg of Heparin (Sigma, H-3149). SFM plus growth factors will be referred to as "growth medium" in the text. Cells were plated onto uncoated tissue culture flasks (Biolite, Fisher Scientific). The neurospheres generated were passaged weekly by mechanical dissociation in growth medium.

### Assessing the effects of IL-10 on NPCs

NPC neurospheres were dissociated into single cells and plated onto PDL-coated multi-chamber glass slides (25,000 cells per chamber) (LabTek II) in SFM. At 1 day following cell plating, the media was changed and an IL-10 dosing assay was performed under the following conditions: (1) control (SFM with no treatment); (2) 10 ng/ml IL-10; (3) 50 ng/ml IL-10; (4) 100 ng/ml IL-10; (5) 200 ng/ml IL-10; (6) 400 ng/ml IL-10. Assessment of NPC proliferation was performed in SFM for 1 day and NPC differentiation in 2% FBS for 7 days as we previously described [3, 36]. For proliferation assessment, bromodeoxyuridine (BrdU 20 μM, Sigma) was added to the cultures 3 h before processing NPCs for immunocytochemistry. IL-10 neutralizing antibody was tested at various concentrations to determine the optimal concentration needed to block IL-10 effects on NPCs.

### Assessing the effects of microglia-conditioned media on NPCs

NPC neurospheres were dissociated into single cells and plated onto PDL-coated multi-chamber glass slides (25,000 cells per chamber) (LabTek II) in SFM. One day following cell plating, the media was changed to 50% NPC SFM and 50% MCM. The following experimental groups were assessed: (1) MCM alone—50% NPC SFM + 50% fresh microglia media; (2) M0—50% NPC SFM + 50% M0 MCM; (3) M1—50% NPC SFM + 50% M1 MCM; (4) M2—50% SFM + 50% M2 MCM. The polarizing factors used to convert microglia to an M1 and M2 phenotype were used as controls at their respective concentrations. IL-10 neutralizing antibody (R&D Systems, MAB417, 0.8 μg/ml) was used to assess the overall effect of IL-10 on NPC proliferation and differentiation. Assessment of NPC proliferation (1 day post MCM treatment) and differentiation (7 days post MCM treatment) was accomplished as described above.

### In vitro immunostaining, image processing, and analysis

For immunocytochemistry, cultures were fixed with 3% paraformaldehyde (PFA) for 20 min at room temperature and washed three times with PBS. Cells were incubated in a blocking solution containing 5% non-fat milk, 1% BSA, and 0.5% Triton X-100 in 0.1 M PBS for 1 h. Cultures then underwent an immunostaining procedure using methods described earlier for immunohistochemistry. For BrdU immunodetection, prior to blocking, sections were washed with PBS, incubated in 2 N HCl and 0.5% Triton X-100 for 30 min at 37 °C, and washed with 0.1 M sodium borate in PBS for 10 min. After blocking, the slides were incubated with primary antibodies overnight and secondary antibodies were added as was previously described. For immunocytochemistry quantification, 8–10 separate fields (under × 20 objective) containing an average of 300 cells for each condition were randomly imaged using a Zeiss AxioObserverZ1 inverted microscope or a Zeiss Imager 2 epi-fluorescence microscope. For each condition, the total number of DAPI-positive cells was first assessed, and the number of positive cells for Olig2, GFAP, and BrdU (containing a DAPI-positive nucleus) were then counted. The percentage of abundance of each cell type was calculated by dividing the number of positive cells for the marker by the total number of DAPI+ cells under each experimental condition. Values were then normalized to control condition for relative comparison.

### Statistical analysis

Using SigmaStat Software (4.0), we performed one-way ANOVA followed by Holm-Sidak post-hoc was used in all Western blot, MPO, MMP, and in vitro analyses. The student *t* test was used when two groups were compared. The data was reported as means ± standard error of the mean (SEM). *P* ≤ 0.05 was considered statistically significant.

## Results

### Inhibition of LAR and PTPσ fosters a beneficial inflammatory response by resident microglia and infiltrating leukocytes following SCI

Recent evidence suggests that degradation of CSPG with ChABC promotes an anti-inflammatory M2 microglia/macrophage response after SCI [27, 28]. Here, we sought to determine the role of LAR and PTPσ receptors in neuroinflammation in rat SCI using functionally blocking peptides against these receptors. We delivered TAT-conjugated ILP and ISP (10 μg each/day) intrathecally to the injured spinal cord in a sustainable manner using an Alzet osmotic pump. The dose of ILP and ISP was previously determined by our group in a contusive model of rat SCI [30]. ILP and ISP treatments were co-delivered in this study as our previous in vitro findings revealed that both receptors mediate CSPG effects and their combinatorial downregulation resulted in the greatest inhibition of CSPG effects in NPCs versus their solitary administration [3]. Infusion of ILP and ISP began immediately after SCI for a duration of 1, 3, 5, 7, or 14 days post-SCI (*N* = 4–6 animals/group/time-point). Stability of ILP and ISP in Alzet osmotic pumps at 37 °C has previously been verified by our group confirming that both ILP and ISP are stable for at least 42 days (data not shown). We performed a battery of tissue assessments to study the impact of LAR and PTPσ receptors on immune response in rat SCI that are described in this section.

#### Neutrophils

**Fig. 2:**
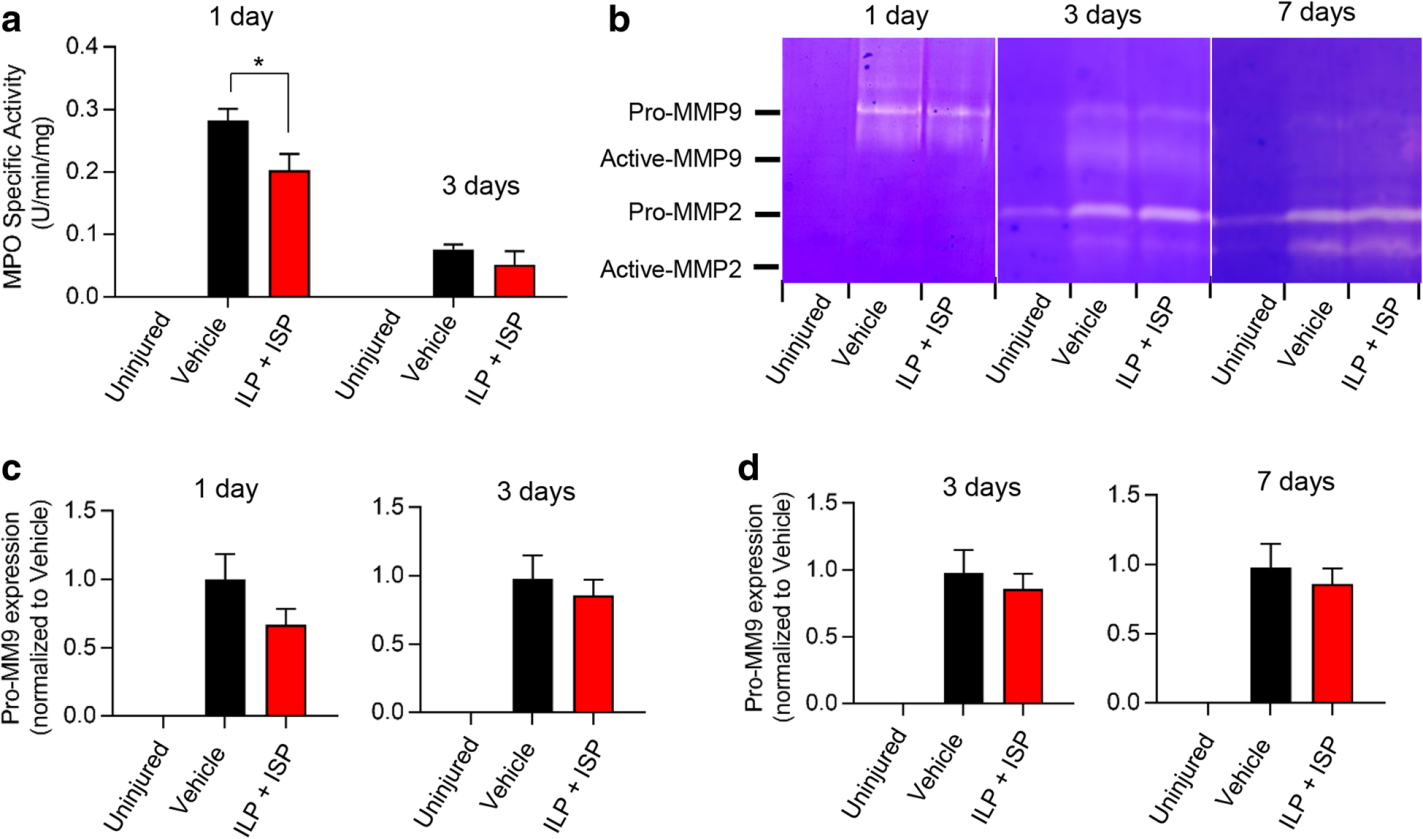
ILP/ISP treatment limits neutrophil infiltration but does not modulate MMP activity in acute SCI. **a** Myeloperoxidase (MPO) activity, a marker for neutrophils, was increased in the injured spinal cord at 1 day following SCI, which was attenuated by ILP/ISP treatment. **b**–**d** Matrix metalloproteinases (MMP)-2 and MMP-9 expression was also assessed using gel zymography. SCI-induced expression of MMP-2 and MMP-9 was observed at 1, 3, and 7 days post-SCI. However, there was no change in the levels of pro-MMP-9 (**c**) and pro-MMP-2 (**d**) under ILP/ISP treatment at any examined time-points. *N* = 4–6 animals/group/time-point. The data show mean ± SEM, *p < 0.05, one-way ANOVA (**a**–**d**)

Neutrophils are the first leukocytes infiltrating the SCI lesion peaking at 24 h post-injury and remaining for up to 3 days [45, 46]. Neutrophil recruitment is associated with a pro-inflammatory phenotype that causes cell death and poor motor recovery [46, 47]. We assessed neutrophil infiltration in SCI by measuring MPO activity, a well-established marker for assessment of neutrophils [38]. While there was no detectable MPO activity in the baseline uninjured group, we found an elevated level of MPO at 1 day post-SCI that was significantly decreased under ILP/ISP treatment suggestive of reduced neutrophil infiltration (Fig. 2a, *N* = 4–6 animals/group/time-point, *p <* 0.05, one-way ANOVA, Holm–Sidak post hoc). At 3 days post-SCI, elevated MPO activity was generally decreased in the injured spinal cord to a level closer to the baseline uninjured group as expected, and there was no difference between vehicle and ILP/ISP treatment groups (Fig. 2a). Additionally, we studied the activity of MMPs in response to ILP/ISP treatment (*N* = 4–6 animals/group/time-point). MMPs are upregulated and activated following SCI and contribute to blood-spinal-barrier degradation and influx of leukocytes into the injured tissue [48]. Using gelatin zymography, we detected a robust increase in MMP-9 at 1 and 3 days and in MMP-2 at 3 and 7 days post-SCI (Fig. 2b–d, *p* < 0.05, one-way ANOVA, Holm–Sidak post hoc). However, ILP and ISP treatment had no apparent effect on modulating the activities of MMP-2 and MMP-9 after SCI in our model.

#### Microglia/macrophages

**Fig. 3:**
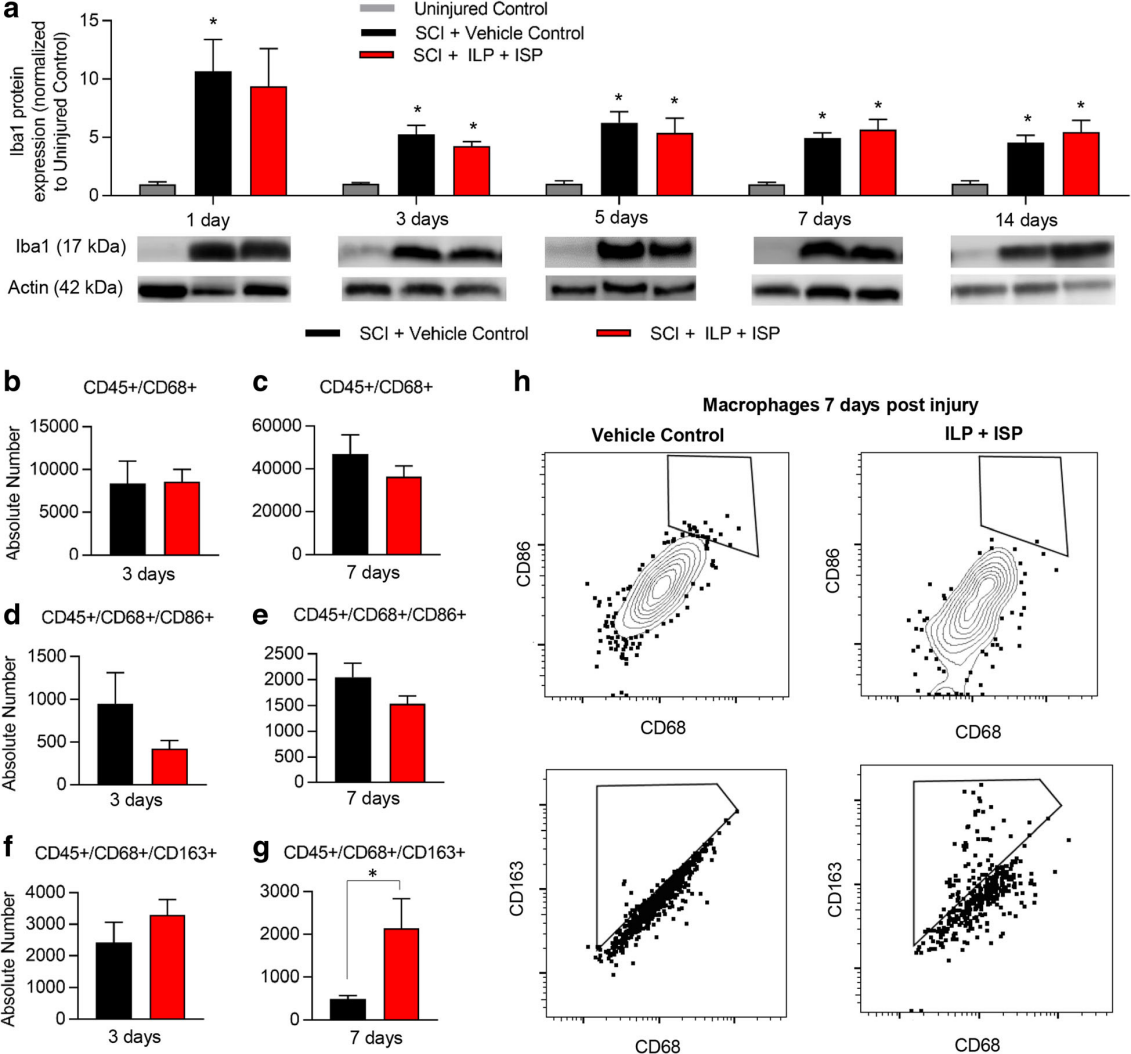
Inhibition of LAR and PTPσ promotes an increase in the subpopulation of M2 macrophages after SCI. **a** Western blot analysis of Iba1 protein expression at 1, 3, 5, 7, and 14 days following SCI revealed no apparent change in the presence of microglia/macrophage within SCI lesion. **b**, **c** Similarly, flow cytometric analysis of spinal cord tissue revealed no change in the total number of infiltrated macrophages (CD45^+^/CD68^+^) at 3 and 7 days post-injury between vehicle control and ILP/ISP (10 μg/day) treated SCI animals. **d**, **e** ILP/ISP treatment resulted in a non-significant decrease in the number of CD45^+^/CD68^+^/CD86^+^ M1 macrophages at 3 and 7 days post-injury. **f**, **g** A significant increase in the number of CD45^+^/CD68^+^CD163^+^ M2 macrophages was observed at 7 days post-injury in ILP/ISP-treated animals. **h** Representative flow cytometry gates are depicted. *N* = 4–6 animals/group/time-point. The data show mean ± SEM, *p < 0.05, one-way ANOVA (**a**), Student *t* test (**b**–**g**)

**Fig. 4:**
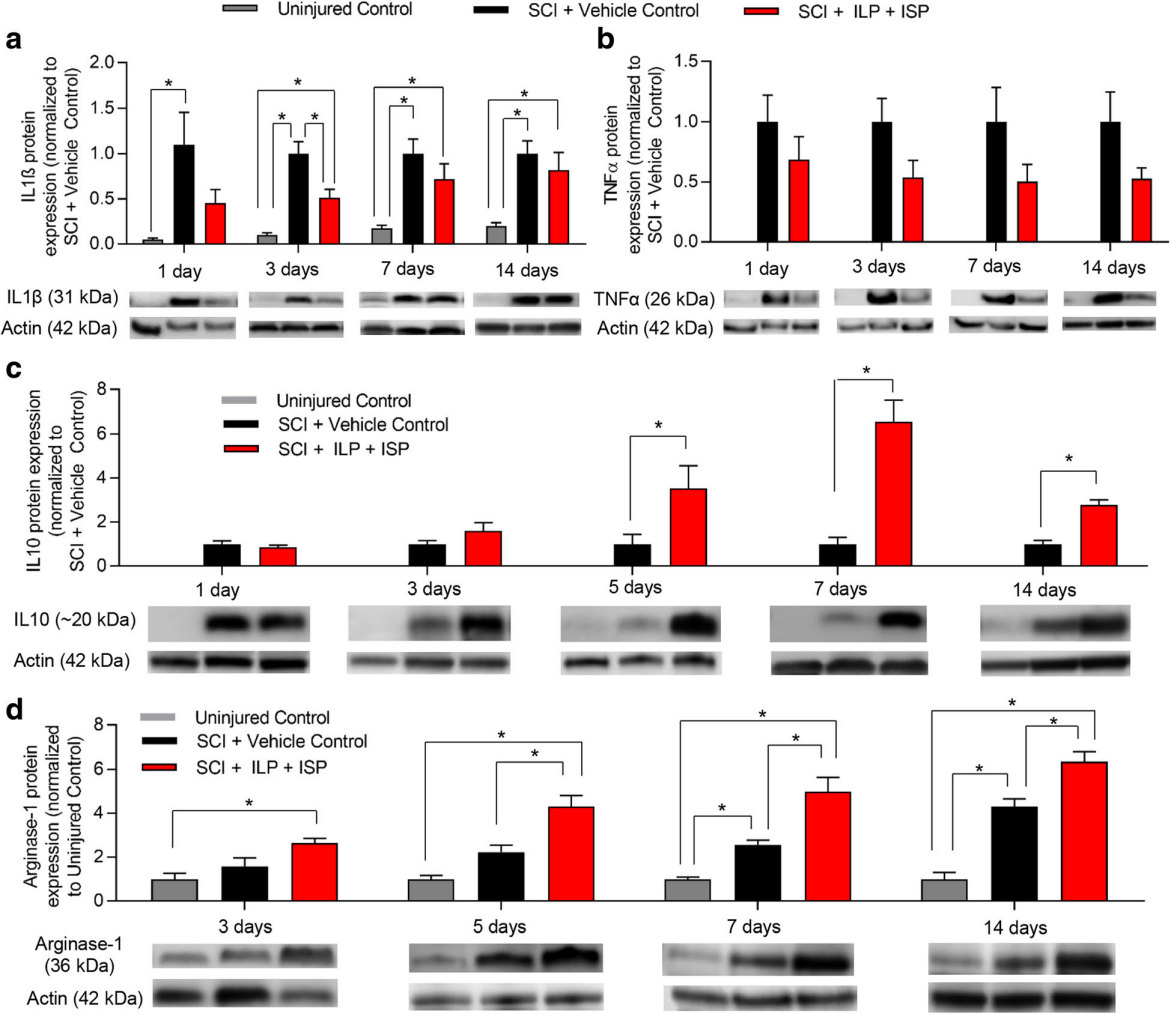
Modulation of LAR and PTPσ attenuates pro-inflammatory cytokines while elevating anti-inflammatory mediators in SCI. **a** Western blot analysis of IL-1β protein expression at 1, 3, 7, and 14 days post-SCI showed a significant increase in IL-1β at 1 day post-SCI that persisted for up to 14 days after injury. ILP and ISP co-treatment attenuated this upregulation but was only statistically significant at the 3-day time-point post-SCI. **b** TNFα protein expression was also significantly upregulated at 1, 3, 7, and 14 days post-SCI compared to uninjured control. ILP and ISP treatment reduced TNFα levels; however, the reduction was not statistically significant. **c**, **d** Western blot analysis of IL-10 and Arginase-1 protein at various time-points showed that ILP and ISP co-treatment significantly increased both factors at 5, 7, and 14 days post-SCI compared to vehicle treatment. *N* = 4–6 animals/group/time-point. The data show the mean ± SEM, *p < 0.05, one-way ANOVA

Resident microglia and infiltrating blood born macrophages are key immune cells that increase their cell numbers in SCI peaking around 7 days post-injury [49–51]. Our analysis of the microglia/macrophages marker Iba-1 at 1, 3, 5, 7, and 14 days post-SCI (*N* = 4–6 animals/group/time-point) revealed that ILP/ISP treatment has no effect on the overall recruitment of microglia/macrophages to the SCI lesion (Fig. 3a, *p* < 0.05, one-way ANOVA, Holm–Sidak post hoc). Similarly, our flow cytometric analysis (*N* = 5 animals/group/time-point) confirmed no apparent change in the overall infiltration of CD45^+^/CD68^+^ macrophages at both 3 and 7 days post-injury (Fig. 3b, c, *p* < 0.05, Student *t* test). Although the number of macrophages remained unaffected, ILP/ISP therapy was able to induce a phenotype shift from a predominantly M1 to a majority M2 profile. We observed a non-significant decrease in the number of CD45^+^/CD68^+^/CD86^+^ M1 macrophages at 3 and 7 days post-injury between vehicle and ILP/ISP-treated animals (Fig. 3d, e, *p* < 0.05, Student *t* test). Conversely, ILP/ISP-treated rats showed a significantly higher number of CD45^+^/CD68^+^/CD163^+^ M2 macrophages in their spinal cord at 7 days post-injury (Fig. 3f, g, *p* < 0.05, Student *t* test). Our cytokine analysis by Western blotting also reaffirmed our flow cytometry data and showed a reduction in pro-inflammatory cytokines, interleukin (IL)-1β and TNFα in ILP/ISP-treated animals as compared to vehicle control group, which was significantly different at the 3-day time-point for IL-1β (Fig. 4a, b, *N* = 4–6 animals/group, *p* < 0.05, one-way ANOVA, Holm-Sidak post hoc). Instead, ILP/ISP treatment resulted in a marked increase in the production of IL-10 and Arginase-1, two well-known M2-associated markers that was statistically significant at 5, 7, and 14 days post-SCI compared to SCI vehicle-treated group (Fig. 4c, d, *p* < 0.05, one-way ANOVA, Holm–Sidak post hoc).

#### T cells

**Fig. 5:**
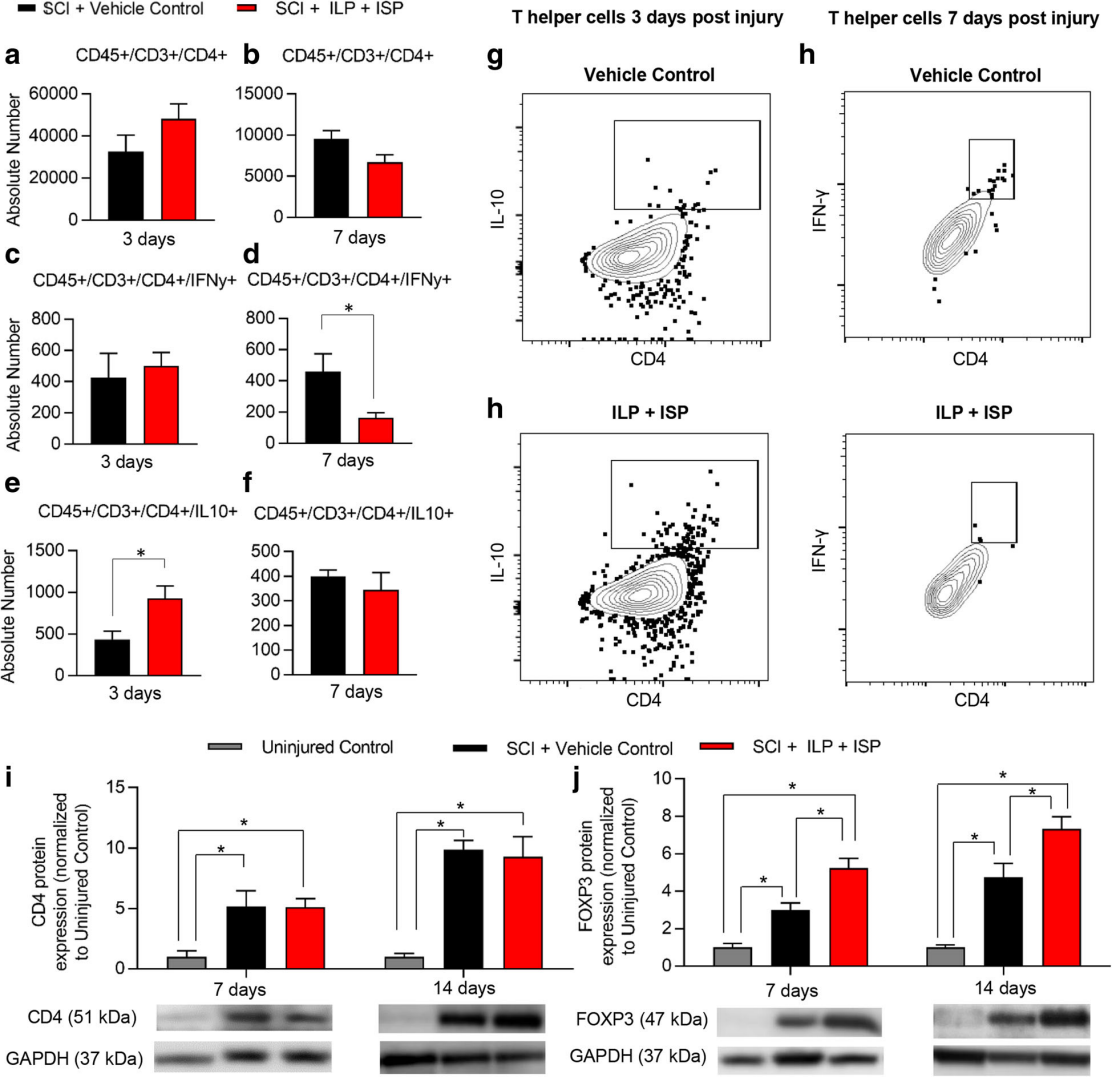
ILP and ISP promotes a phenotypic switch in helper T cells toward a T_reg_ phenotype. **a**, **b** Flow cytometric assessment revealed no apparent difference in the overall infiltration of helper T cells (CD45^+^/CD3^+^/CD4^+^) in the injured spinal cord at 3 and 7 days post-injury between vehicle and ILP/ISP-treated animals. **c**, **d** However, ILP/ISP-treated animals exhibited a significant decrease in the number of effector T cells (CD45^+^/CD3^+^/CD4^+^/IFNγ^+^) at 7 days post-injury. **e**, **f** A significant increase in the total number of regulatory T cells (CD45^+^/CD3^+^/CD4^+^/IL10^+^) was observed at 3 days post-injury in ILP/ISP-treated animals. **g**, **h** Representative flow cytometry gates are shown. **i** Western blot analysis showed upregulation of CD4 protein expression, at 7 and 14 days post-SCI compared to uninjured control group confirming infiltration of helper T cells in the injured spinal cord. Confirming our flow cytometry, ILP and ISP had no apparent effect on the overall protein expression of CD4. **j** However, ILP and ISP significantly increased FOXP3 protein expression, a marker of regulatory T cells, at both 7 and 14 days post-SCI compared to SCI vehicle control. Western blot results have been normalized to the actin loading control prior to subsequent normalization to the control values. The data show mean ± SEM, *p < 0.05, Student *t* test (**a**–**f**), one-way ANOVA (**i**, **j**), N = 4-6/group

**Fig. 6:**
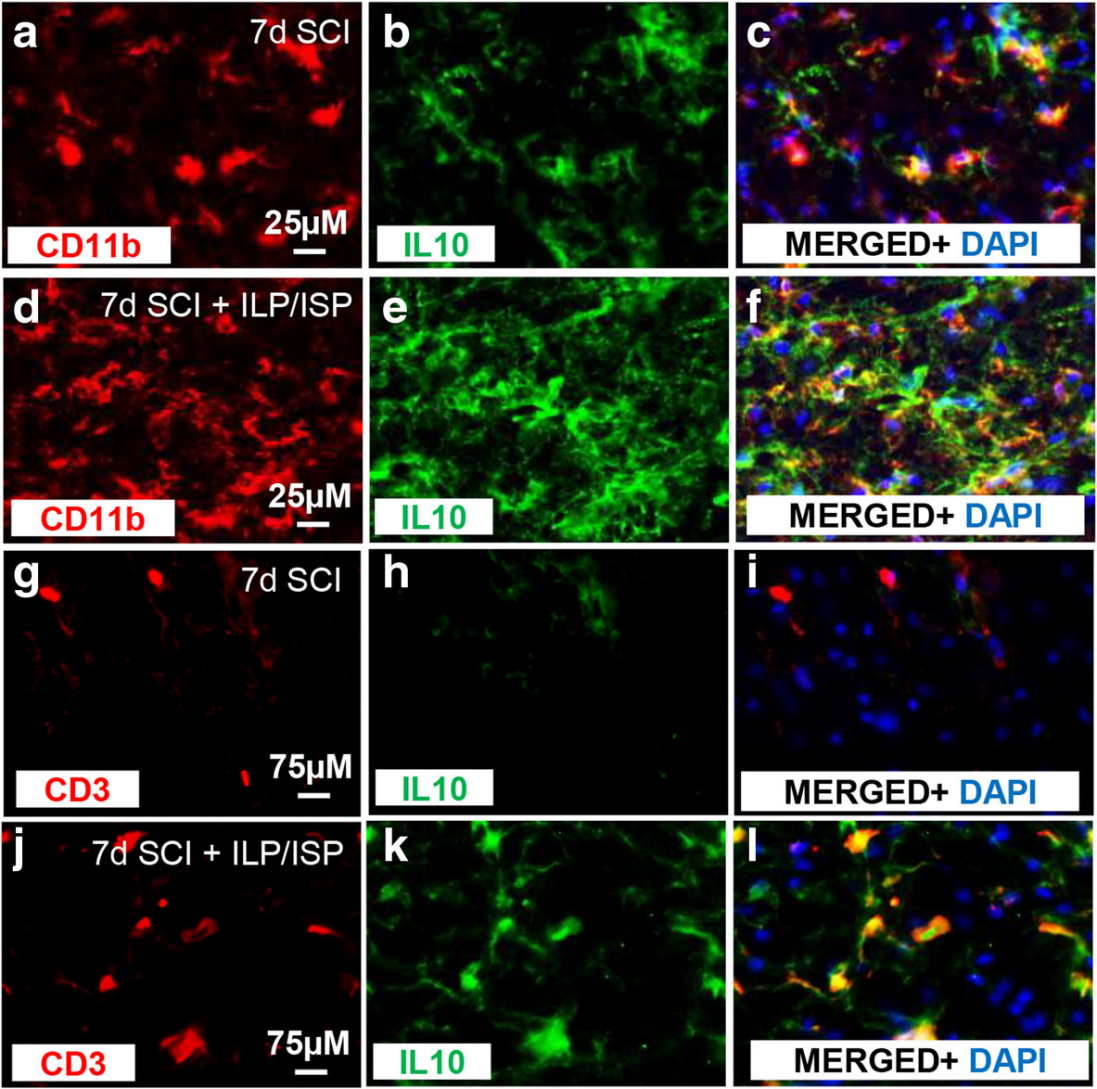
Microglia/macrophages and T cells contribute to IL-10 expression after SCI. **a**–**l** Immunohistochemistry on spinal cord tissue confirmed an increase in IL-10 expression in ILP/ISP-treated animals at 7 days following injury compared to vehicle-treated animals. IL-10 expression was confirmed to be expressed in both CD11b + macrophages/microglia (**a**–**f**) and CD3^+^ T cells (**g**–**l**)

Given that inhibition of LAR and PTPσ promoted a remarkable IL-10 response, we next studied infiltrating regulatory T (T_reg_) lymphocytes that also produce IL-10 in the injured spinal cord. Our flow cytometric analysis showed no apparent change in the number of CD45^+^/CD3^+^/CD4^+^ helper T cells with ILP/ISP treatment at both 3 and 7 days post-injury (Fig. 5a, b, *N* = 5 animals/group, *p <* 0.05, Student *t* test). Western blot analysis also confirmed our flow cytometry data showing no change in the overall number of CD4^+^ helper T cells between ILP/ISP and vehicle-treated animals at 7 and 14 days post-injury (Fig. 5i, *p* < 0.05, one-way ANOVA, Holm–Sidak post hoc). However, similar to macrophages, blockade of LAR and PTPσ promoted a phenotype change in T cells. ILP/ISP treatment led to a significant reduction in the number of IFNγ expressing effector T cells (CD45^+^/CD3^+^/CD4^+^/IFNγ^+^) at 7 days post-injury (Fig. 5c, d, *p* < 0.05, Student *t* test) while promoting a significant increase in IL-10 expressing T_reg_ cells (CD45^+^/CD3^+^/CD4^+^/IL10^+^) at 3 days post-injury (Fig. 5e, f, *p* < 0.05, Student *t* test). Moreover, our complementary Western blot analysis also identified a significant increase in forkhead box P3 (FOXP3) protein expression, a marker associated with IL-10 expressing T_reg_ cells, at both 7 and 14 days post-injury (Fig. 5j, *p* < 0.05, one-way ANOVA, Holm–Sidak post hoc). Interestingly, our immunohistochemical analysis of spinal cord tissue at 7 days post-SCI confirmed that both CD11b microglia/macrophages (Fig. 6a–f) and CD3 T cells (Fig. 6g–l) contribute to IL-10 expression following SCI, and this expression was elevated in ILP/ISP-treated animals.

### CSPGs negatively modulate microglia responses by signaling through LAR and PTPσ receptors and activation of the Rho/ROCK pathway

**Fig. 7:**
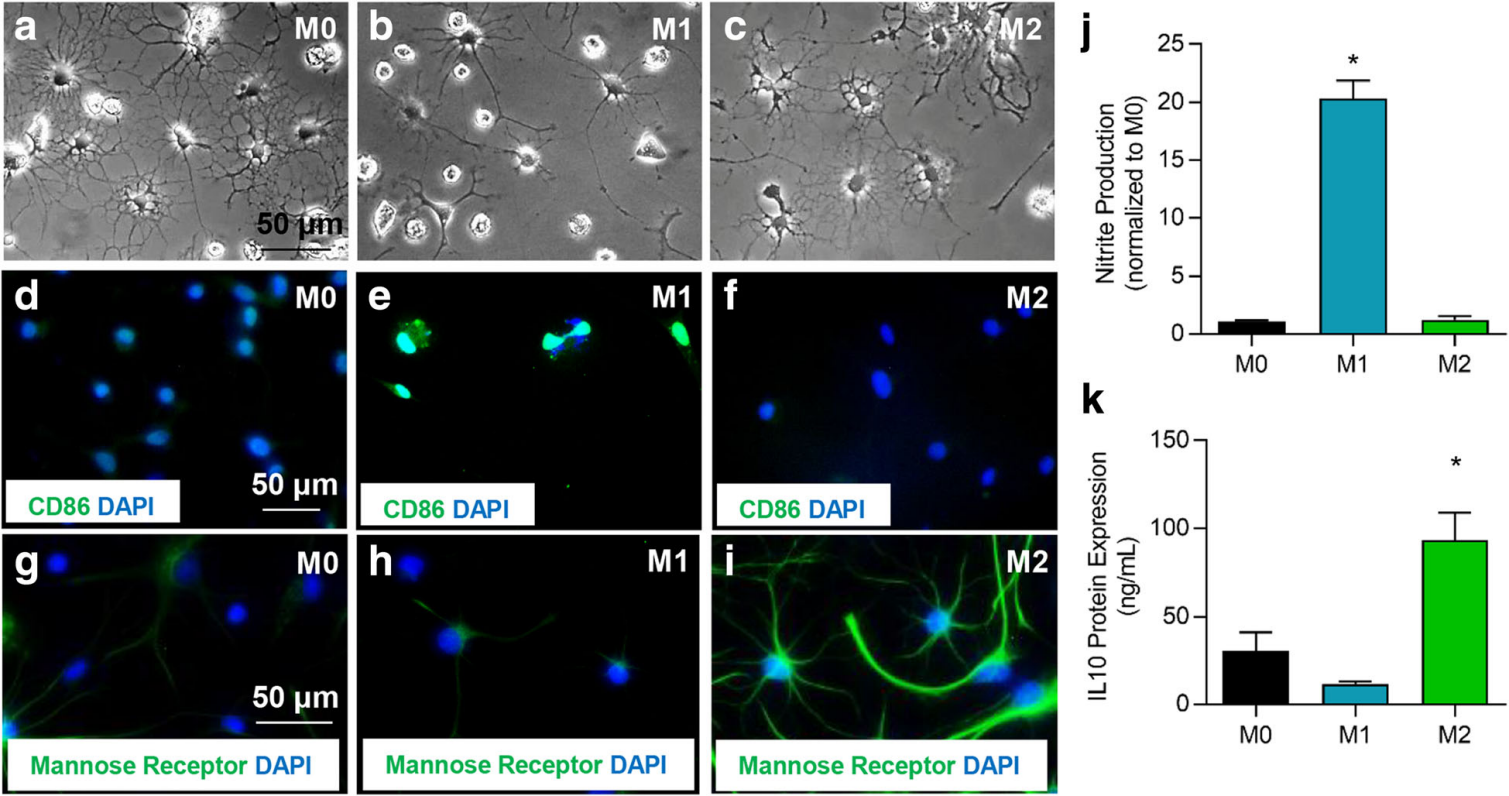
Polarization of primary microglia cultures to an M0, M1, or M2 phenotype. **a**–**c** Primary microglia were polarized to M1 through IFNγ and TNFα treatment or M2 through IL-10 treatment. M1 polarization was confirmed by induced expression of CD86 (**d**–**f**) and release of nitrite (**j**). Increased expression of mannose receptor (**g**–**i**) and IL-10 (**k**) were used to confirm M2 polarization. *N* = 5 independent experiments. The data show the mean ± SEM, *p < 0.05, one-way ANOVA

Our SCI studies demonstrated that inhibition of CSPGs receptors, LAR and PTPσ, promoted an M2 phenotype. Thus, we next dissected the role and mechanisms of CSPGs in regulating the microglia response and whether LAR and PTPσ mediate CSPG effects using a direct in vitro primary microglia culture model. First, we confirmed that microglia express both LAR and PTPσ (Additional file 5: Figure S5A-F). Next, we determined the direct effects of CSPGs on several aspects of microglia functions including phagocytosis, nitrite production, cytokine release, and mobilization in the various microglia phenotypes including resting (M0), classically activated (M1), and alternatively activated (M2). Microglia were polarized to an M1 phenotype by co-treatment of IFNγ (50 ng/ml) and TNFα (40 ng/ml) or an M2 phenotype by IL-10 (10 ng/ml) treatment (Fig. 7a–c). M1 polarization was confirmed by induced expression of CD86 (Fig. 7d–f) and nitric oxide (Fig. 7j, *N* = 5 of independent cultures, *p* < 0.05, one-way ANOVA, Holm–Sidak post hoc) that was absent in M0 (untreated microglia) or M2 microglia. Similarly, M2 microglia polarization was confirmed by an increase in the mannose receptor (Fig. 7g–i) and IL-10 production (Fig. 7k, *N* = 5 of independent cultures *p* < 0.05, one-way ANOVA, Holm–Sidak post hoc), two known markers of the M2 dominant phenotype [24].

#### Microglia phagocytosis

**Fig. 8:**
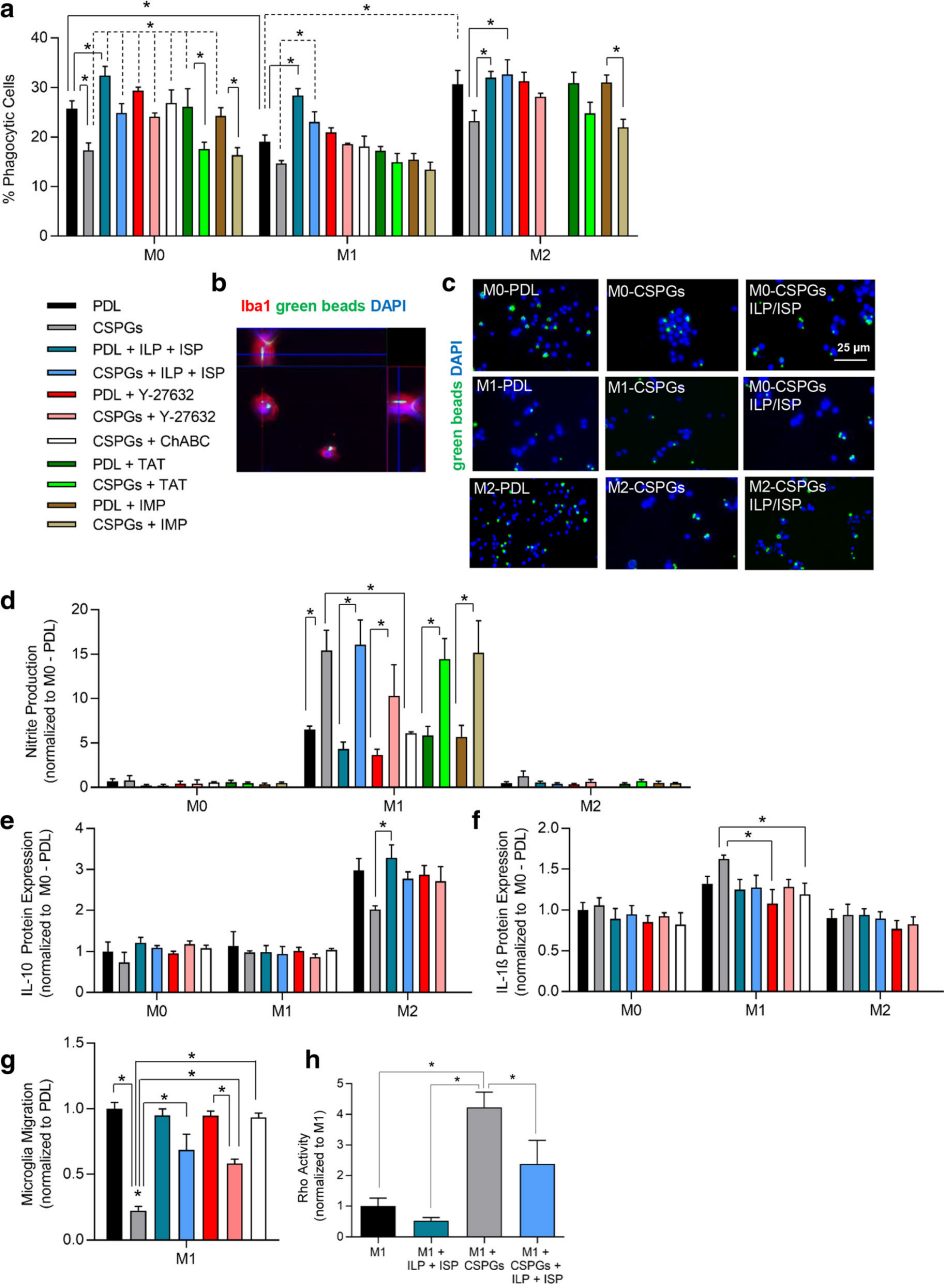
CSPGs modulate microglia phagocytosis, migration, and nitrite production which is partially mediated through LAR and PTPσ signaling and Rho activation. **a**, **b** Microglia phagocytosis was assessed. Success of phagocytosis was verified by intracellular detection of green fluorescent beads in microglia (Iba-1+) through Z-stack imaging. M1 microglia (TNFα + IFNγ treated) showed a reduced ability for phagocytosis. CSPGs reduced phagocytosis in microglia, which was attenuated and even promoted with ILP/ISP, inhibition of ROCK by Y-27632, or ChABC treatment but not by TAT or IMP control peptides. **c** Representative images of phagocytosis by M0, M1, and M2 microglia are depicted for PDL, CSPGs, and CSPGs + ILP/ISP conditions. **d** Nitrite production was exacerbated and significantly increased in M1 microglia when exposed to CSPG. This effect was not blocked by ILP/ISP, Y-27632, IMP, or TAT treatment but was blocked by ChABC degradation of CSPGs. IL-10 (**e**) and IL-1β (**f**) release was assessed in microglia 2 days after plating onto PDL or PDL + CSPGs substrate. CSPGs reduced IL-10 expression in M2 microglia while had no significant effect on IL-1β release. **g** CSPGs also significantly limited microglia migration which was overcome by ILP/ISP, Y-27632, and ChABC treatment. **h** RhoA activity was assessed by G-LISA in microglia demonstrating a significant increase in Rho activity when microglia were exposed to CSPGs substrate. ILP and ISP treatment significantly decreased Rho activity. The data show the mean ± SEM, *p < 0.05, one-way ANOVA, *N* = 3–5/group. *N* = 3–5 independent experiments. The data show the mean ± SEM, *p < 0.05, one-way ANOVA

In cultures of M0, M1, and M2 polarized microglia, we first analyzed the effects of CSPGs on modulating microglia phagocytic ability (Fig. 8a). M2 microglia/macrophages are responsible for the clearance of degenerating cells and myelin debris in SCI, and thereby contribute to tissue repair [18]. Green fluorescent beads were pre-opsonized with serum and then added to microglia grown on PDL or PDL + CSPGs substrate (Fig. 8b, c). Presence of CSPGs significantly decreased M0 phagocytosis by 29% which was overcome by both ILP/ISP treatment and degradation of CSPGs with ChABC (Fig. 8a, *N* = 3–4 independent cultures, *p <* 0.05, one-way ANOVA, Holm–Sidak post hoc). Interestingly, treating microglia with the ROCK inhibitor Y-27632 (10 μM) was also able to attenuate CSPG effects on microglia phagocytosis indicating a Rho/ROCK-mediated mechanism. Notably, in M0 “resting” microglia, ILP/ISP treatment itself significantly increased microglia phagocytosis compared to PDL control group. TAT and IMP control peptides did not appear to have any modulatory effect on microglia phagocytosis on their own. M1 microglia showed reduced phagocytosis ability compared to M0 microglia, but CSPGs did not further decrease their phagocytic ability. Similar to M0 microglia, ILP/ISP treatment significantly promoted M1 microglia phagocytosis in both PDL and PDL + CSPGs-treated cells suggesting the overall inhibitory role of LAR and PTPσ in phagocytosis independent of CSPGs. Our analysis revealed that M2 polarization significantly enhanced the ability of microglia for phagocytosis as compared to M1 counterparts in the baseline PDL condition. Exposure to CSPGs non-significantly decreased phagocytosis ability of M2 microglia as compared to PDL condition, which was entirely reversed by ILP/ISP treatment. Interestingly, similar to M0 and M1 microglia, ILP/ISP treatment promoted the ability of M2 microglia for phagocytosis in the presence of CSPGs. However, in contrast to M0 and M1 microglia, ILP/ISP treatment on its own had no apparent effects on the baseline level of phagocytosis in M2 microglia in the PDL condition without CSPGs.

#### Assessment of microglial phenotypes

We further investigated whether CSPGs induce an M1 pro-inflammatory state in microglia. To this end, we studied nitrite production of microglia as an indicator of the M1 phenotype [18]. M0 and M2 microglia released rather low levels of nitrite, which remained unaffected by the presence of CSPGs indicating that CSPGs themselves do not induce an M1 state in M0 “resting” or M2 microglia (Fig. 8c, *N* = 3–4, *p* < 0.05, one-way ANOVA, Holm–Sidak post hoc). However, exposure of M1 cells to CSPGs exacerbated their nitrite production compared to M1 microglia grown on PDL. Interestingly, this effect was not blocked by ILP and ISP treatment or Y-27632, while ChABC did block CSPGs-induced nitrite production in M1 cells. These results indicate that a CSPG-dependent mechanism exists for nitrite release in M1 microglia that is not mediated by LAR and PTPσ or Rho/ROCK pathways. TAT and IMP control peptides also had no apparent effect on microglia nitrite levels.

We then examined whether CSPGs modulate cytokine release by microglia. We studied IL-1β and IL-10 representing the M1 and M2 dominant phenotype, respectively. Overall, there was no apparent change in IL-10 or IL-1β expression in M0 “resting” microglia when grown on PDL or PDL + CSPG substrate (Fig. 8d, e, *N* = 3–4 independent cultures, *p* < 0.05, one-way ANOVA, Holm–Sidak post hoc). CSPGs did however non-significantly decrease IL-10 expression in M2 microglia which was brought to control levels with ILP/ISP, Y-27632, and ChABC treatment. Conversely, IL-1β expression was non-significantly increased when M1 microglia were exposed to the CSPG substrate. Given the involvement of Rho/Rock pathway in CSPG effects, we sought to determine whether LAR and PTPσ receptors mediate these effects in microglia cultures. M1 microglia grown on a CSPG substrate had a fourfold increase in Rho activity that was significantly reduced by ILP/ISP (Fig. 8g, *N* = 3 independent cultures, *p* < 0.05, one-way ANOVA, Holm–Sidak post hoc). These findings collectively identify Rho/ROCK pathway as a downstream mediator of CSPGs and LAR and PTPσ signaling.

#### Microglial mobilization

Mobilization of resident microglia to the site of injury is an important aspect of neuroinflammatory processes. Therefore, we investigated whether CSPGs influence the ability of microglia for mobilization using C5a as a chemoattractant in a transwell assay [44]. We quantified the number of microglia which crossed over PDL or PDL + CSPG-coated inserts in a 16-h period. CSPGs significantly decreased microglia mobilization, which was partially yet significantly attenuated by both ILP/ISP and Y-27632 treatments in a comparable manner (Fig. 8f, *N* = 3 independent cultures, *p* < 0.05, one-way ANOVA, Holm–Sidak post hoc). Interestingly, degradation of CSPGs with ChABC was able to block their inhibitory effects on microglia mobilization entirely, suggesting involvement of other mechanisms. Taken together, these data indicate that CSPGs modulate several facets of microglia activity by signaling through LAR and PTPσ and activation of the Rho/ROCK pathway.

### M2 microglia promote oligodendrogenesis by NPCs through an IL-10-mediated mechanism

Emerging evidence has implicated M2 inflammatory response and specifically increased IL-10 release in enhancing endogenous repair mechanisms following central nervous system (CNS) injury [18, 23, 24, 52]. M2 microglia have been shown to promote oligodendrocyte differentiation and myelination in lysolecithin demyelinating mouse models [24]. Interestingly, previous findings by our group and others have revealed that inhibition of CSPGs can also enhance oligodendrogenesis in SCI and demyelinating conditions [5, 53, 54]. Here, we investigated whether the immunomodulatory benefits of inhibiting LAR and PTPσ in promoting an M2 phenotype and IL-10 production can foster oligodendrocyte differentiation. We addressed this question using primary cultures of adult spinal cord-derived NPCs.

**Fig. 9:**
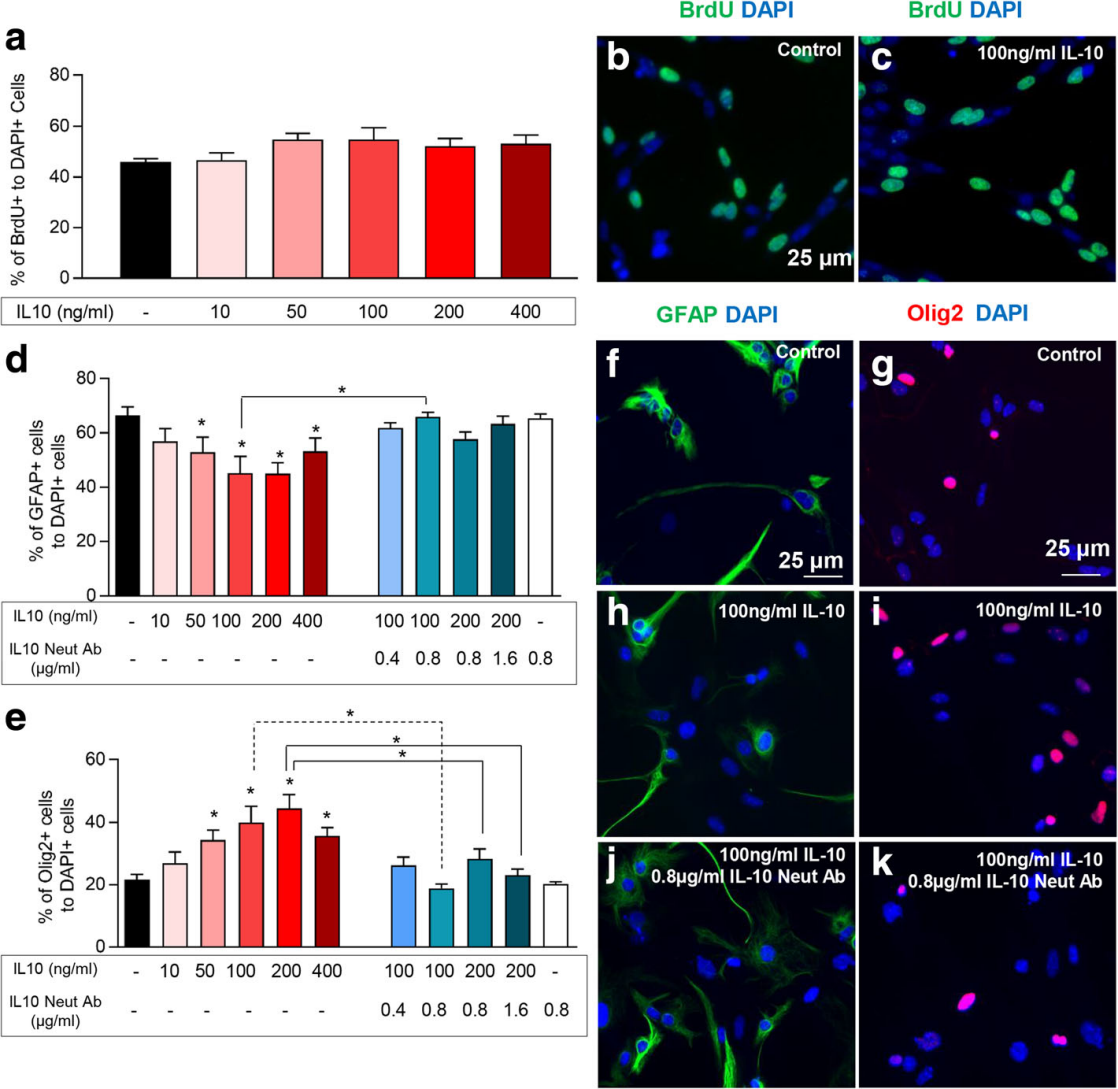
IL-10 promotes oligodendrocyte differentiation of spinal cord NPCs in vitro*.* **a–c** Addition of recombinant IL-10 had no apparent effect on NPCs proliferation (BrdU+/DAPI+) at all doses tested. **d** However, IL-10 significantly reduced the percentage of GFAP+/DAPI+ astrocytes at 100 and 200 ng/ml, **e** while increased the percentage of Olig2+ cells at 50, 100, 200, and 400 ng/ml with the highest effect at 200 ng. This effect was significantly attenuated with IL-10 neutralizing antibody. Addition of 0.8 μg/ml of IL10 neutralizing antibody effectively blocked 100 ng/ml of IL-10 on astrocyte and oligodendrocyte differentiation of NPCs. **f**–**k** Representative images of NPC differentiation assessment are shown. *N* = 3–5 independent experiments. The data show the mean ± SEM, *p < 0.05, one-way ANOVA

First, we assessed whether an increase in the production of IL-10 can directly impact the regenerative response of NPCs by utilizing recombinant IL-10 in vitro. We treated NPCs with recombinant IL-10 and studied their proliferation and differentiation patterns. Assessing the percentage of BrdU+ proliferating NPCs under various concentrations of IL-10 showed no apparent effect on NPC proliferation (Fig. 9a–c, *N* = 5 independent cultures, *p* < 0.05, one-way ANOVA, Holm–Sidak post hoc). However, IL-10 had a modulatory effect on the differentiation pattern of NPCs. IL-10 treatment significantly reduced the number of NPC-derived GFAP+ astrocytes while increasing Olig2+ oligodendrocytes at 100 and 200 ng, which was blocked using 0.8 and 1.6 μg of IL-10 neutralizing antibody, respectively (Fig. 9d–k, *N* = 4, *p* < 0.05, one-way ANOVA, Holm–Sidak post hoc). These direct analyses indicate that availability of IL-10 can promote NPCs differentiation along an oligodendrocyte lineage with no apparent effects on their proliferation.

**Fig. 10:**
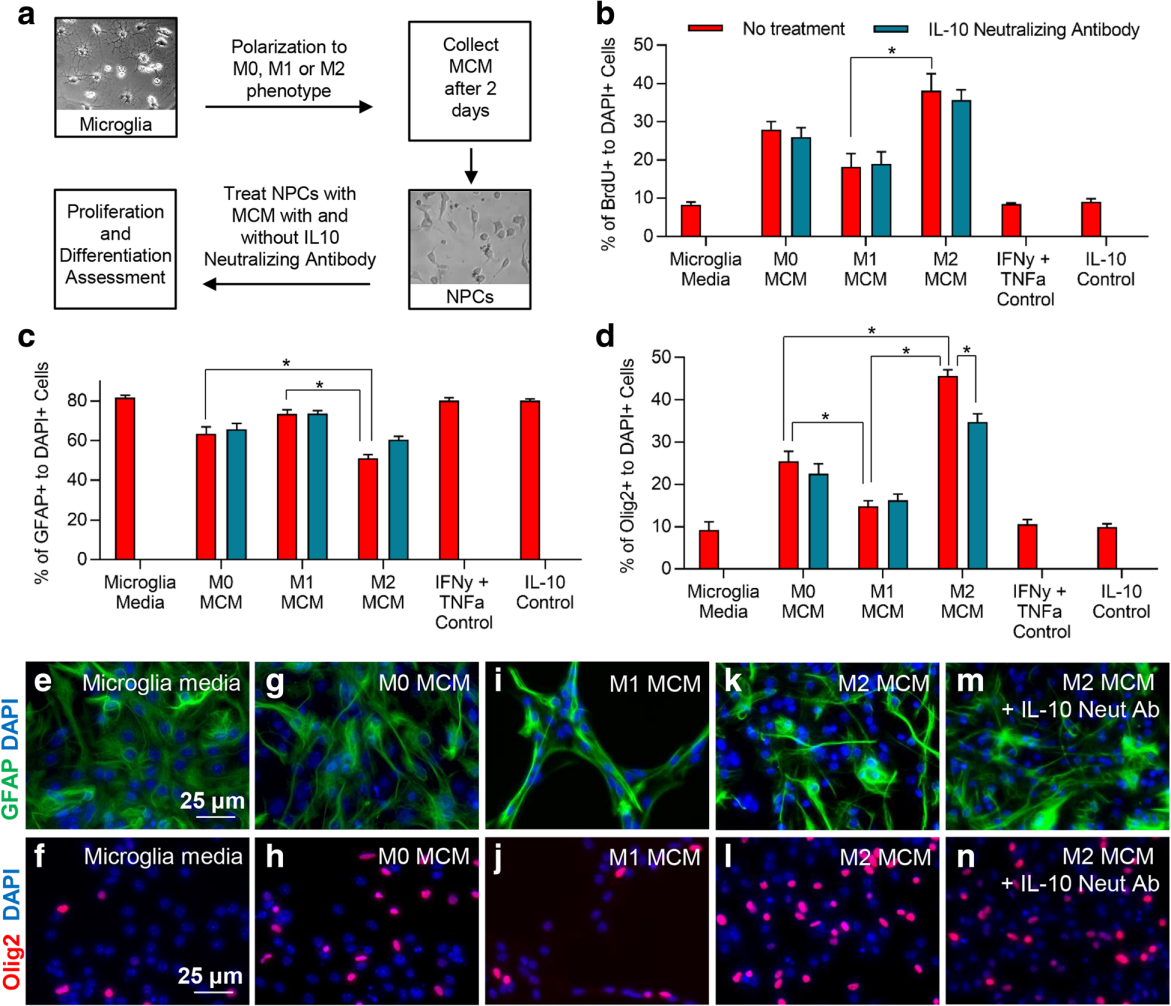
M2 microglia promote oligodendrocyte differentiation of spinal cord NPCs through IL-10. **a** To assess the effects of microglia effects on NPC proliferation and differentiation, MCM was collected from microglia 2 days following polarization. This media was then transferred to NPC cultures to assess proliferation and differentiation. **b** MCM derived from M2 microglia significantly promoted NPC proliferation compared to M1 MCM. IL-10 neutralizing antibody had no effect on the overall proliferation of NPCs by M2 MCM suggesting this effect was not mediated through IL-10. **c** A significant decrease was observed in the percentage of GFAP+/DAPI+ astrocytes when NPCs were exposed to M2 MCM (**k**) compared to both M0 (**g**) and M1 (**i**) MCM. (**d**) M2 MCM (**l**) significantly increased the percentage of Olig2+/DAPI+ cells compared to both M0 (**h**) and M1 (**j**) MCM. Additionally, M1 MCM significantly reduced the percentage of Olig2+ cells compared to both M0 and M2 MCM. **m**, **n** The effect of M2 MCM was significantly reduced by IL-10 neutralizing antibody. **e, f** represents NPCs *N* = 3–5 independent experiments. The data show the mean ± SEM, *p < 0.05, one-way ANOVA

Next, we focused on dissecting the impact of IL-10 derived from M2 polarized microglia on the regenerative response of spinal cord NPCs. Microglia were polarized to an M1 or M2 phenotype as previously described. Two days following microglial polarization, microglia-conditioned media (MCM) was harvested and added to NPC cultures for proliferation and differentiation assessments (Fig. 10a). To assess microglial-derived IL-10 effects on NPCs in vitro*,* we blocked IL-10 using a neutralizing antibody. To estimate the IL-10 protein concentration in the MCM of M2 microglia, our ELISA analysis determined a concentration of 100 ng/ml for IL-10 in MCM (*N* = 5, Fig. 7k). Therefore, 0.8 μg/ml of IL-10 neutralizing antibody was used as this dose was shown to sufficiently block the effects of 100 ng/ml of IL-10 on NPCs oligodendrocyte differentiation in vitro (Fig. 9d, e). Using these parameters, we found that M2 MCM enhances the overall proliferation of NPCs, whereas M1 MCM reduces their capacity for proliferation (Fig. 10b, *N* = 4, *p* < 0.05, one-way ANOVA, Holm–Sidak post hoc). Nonetheless, this effect was found to be IL-10 independent indicating that other factors associated with M2 microglia attribute to the increase in NPC proliferation. Our NPC differentiation studies showed that M2 MCM also promotes a significant increase in oligodendrogenesis of NPCs compared to M0 and M1 MCM (Fig. 10c–n, *N* = 4). Interestingly, this effect was partly attributed to M2-derived IL-10 since an IL-10 neutralizing antibody was able to reverse some of the effect. Conversely, M1 MCM significantly reduced the percentage of NPC-derived oligodendrocytes compared to M0 and M2 MCM (*p* < 0.05, one-way ANOVA, Holm–Sidak post hoc). Astrocyte differentiation of NPCs was decreased by M2 MCM in comparison to M0 and M1 MCM suggesting that M2 polarization supports oligodendrogenesis of spinal cord NPCs. Taken together, these data demonstrate the pivotal role of microglia in regulating endogenous cell differentiation.

### Inhibition of LAR and PTPσ has no effects on CSPG expression and scar formation in SCI

**Fig. 11:**
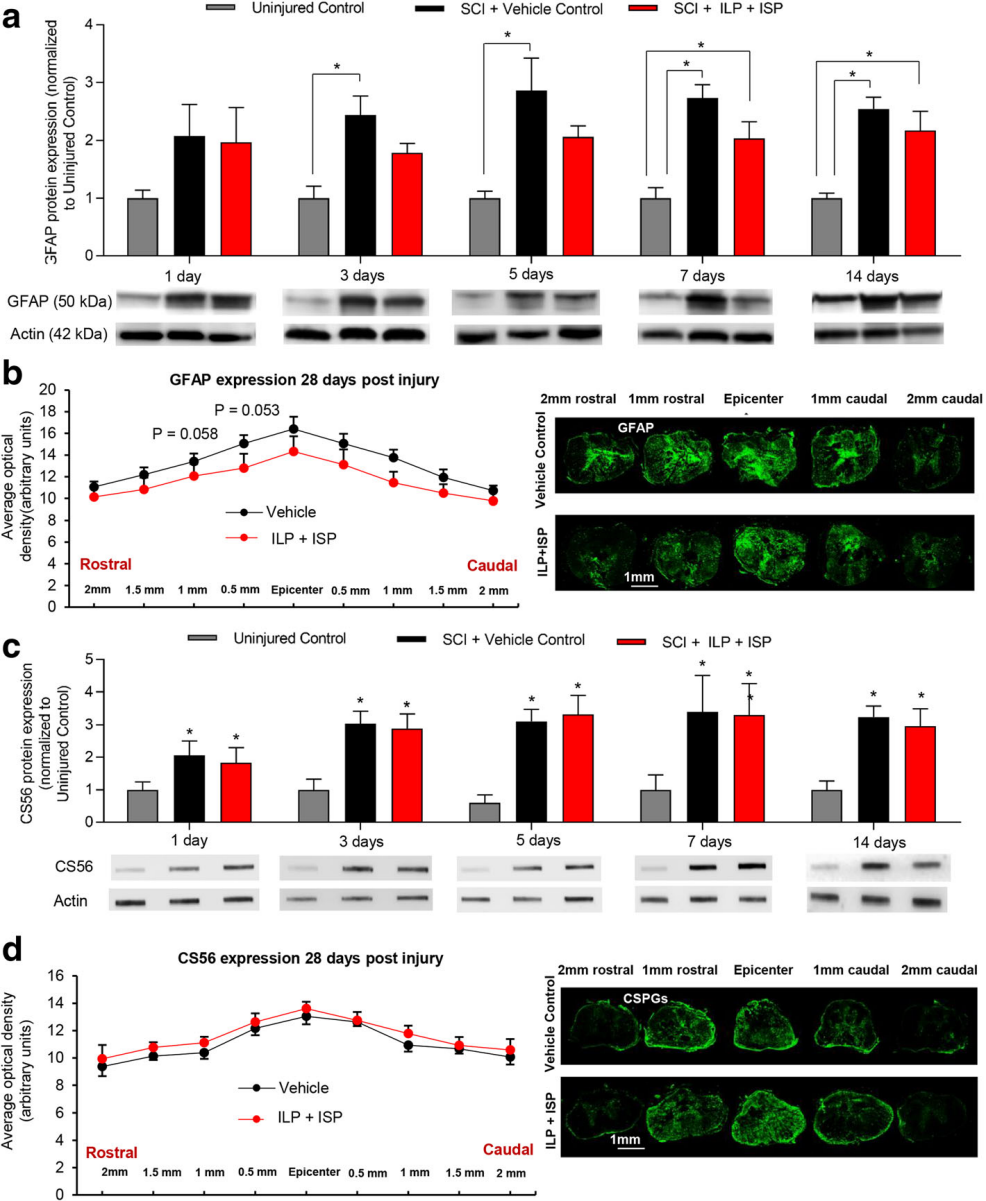
Blocking LAR and PTPσ receptors has no effect on formation of astrocytic scar and CSPGs following SCI. **a** To study scar formation under ILP and ISP treatment, we examined GFAP protein expression by Western blotting at 1, 3, 5, 7, and 14 days post-injury. As anticipated, GFAP protein expression was significantly increased following injury compared to uninjured animals. However, ILP and ISP treatment had no significant effect on the expression of GFAP at any time-points after SCI. **b** Using immunohistochemistry, we also studied astrocytic scar in SCI lesion. Our quantitative immunodensity analysis in the injured spinal cord tissue at the chronic 28 days post-SCI also showed no significant differences in the levels of GFAP under ILP/ISP treatment compared to vehicle SCI control group. **c** Slot blot analysis of CS56 expression at 1, 3, 5, 7, and 14 days post-SCI demonstrated a significant increase CSPGs following injury. Similar to GFAP, ILP and ISP treatment had no effect on the deposition of CSPGs following injury. **d** CS56 expression was additionally measured using immunohistochemistry at different distances to injury epicenter at 28 days post-SCI. ILP/ISP treatment showed no significant differences in the levels of CS56 compared to vehicle SCI control group. The data show the mean ± SEM, *p < 0.05, **p < 0.001, one-way ANOVA (**a**, **c**), two-way ANOVA (**b**–**d**), *N* = 4–6/group

Lastly, we asked whether LAR and PTPσ control CSPGs expression and scar formation following injury through self-regulatory mechanisms. Immunoblotting for GFAP and CSPGs at 1, 3, 5, 7, and 14 days following SCI showed no significant difference in GFAP and CSPGs protein expression between ILP/ISP and vehicle-treated animals at any examined time-point (*N* = 4–6 animals/group, Fig. 11a, c). Similarly, our immunohistochemical measurement of GFAP and CSPGs within the SCI lesion at 7 days (data not shown) and 28 days post-injury suggested no significant difference between vehicle and ILP/ISP treatment groups (Fig. 11b, d, *N* = 5–6 animals/group). As expected, SCI induced an anticipated significant increase in CSPGs and GFAP levels compared to uninjured baseline at all time-points post-injury; however, ILP/ISP treatment did not alter their expression. Altogether, these findings suggest that LAR and PTPσ are not apparently involved in astrocytic scar formation and CSPG deposition in the injured spinal cord.

## Discussion

### Overview of findings

In the present study, we have identified novel inhibitory mechanisms for CSPGs and their specific signaling receptors, LAR and PTPσ, in modulating the immune response after SCI. In a clinically relevant model of compressive/contusive SCI in rat, we demonstrate that modulation of CSPG signaling with ILP and ISP treatment drives an anti-inflammatory and pro-regenerative immune response that is characterized by increased populations of M2 microglia/macrophages and T regulatory cells within the injured spinal cord. This cellular response was associated with an overall decrease in pro-inflammatory markers including IL-1β and TNFα and an increase in regulatory mediators such as IL-10, Arginase-1, and FOXP3. Our complementary in vitro studies in primary microglia revealed that while CSPGs do not seem to be an inducer of the M1 phenotype in microglia per se, their presence in the milieu of M1 microglia promotes and/or maintains their pro-inflammatory phenotype. We demonstrate that CSPGs promote production of M1 markers IL-1β and nitrite in microglia. Interestingly, while the CSPG-induced increase in IL-1β was attenuated by ILP/ISP, nitrite production was not mediated by LAR and PTPσ signaling. This evidence suggests that CSPGs seem to regulate nitrite production in M1 microglia through other mechanisms, which needs further elucidation. Interestingly, the presence of CSPGs also suppressed IL-10 release by M2-polarized microglia, which was ameliorated by inhibition of LAR and PTPσ signaling. Importantly, we provide novel direct evidence that CSPGs, through LAR and PTPσ activation, hinder the ability of microglia for phagocytosis and mobilization, and suppress their potential for promoting oligodendrocyte differentiation of NPCs. Mechanistically, we demonstrate that LAR and PTPσ mediate the effects of CSPGs on microglia through RhoA activation and the ROCK pathway. Altogether, our parallel findings in SCI and microglial cultures have uncovered, for the first time, a negative immunomodulatory role for CSPGs and the LAR/PTPσ axis that can contribute to the poor regenerative response after SCI. Thus, we propose that targeting LAR and PTPσ represents a potential immunotherapy strategy for SCI.

### Inhibition of LAR and PTPσ positively regulates inflammatory processes after SCI

Neuroinflammation is a complex process in SCI that involves several cell types including resident microglia and infiltrating leukocytes [49, 50]. The first phase of neuroinflammation involves recruitment of resident microglia and neutrophils. Neutrophils are the first leukocyte population to enter the spinal cord after insult [45, 46, 55]. Neutrophils are recruited to the injury site within 6 h after injury and their numbers peak within 24 h [45, 46]. They are generally thought to exacerbate the injury process by damaging neurons, glial cells, and endothelial cells, and through their release of toxic ROS and proteases [56, 57]. Here, we demonstrate the ability of ILP and ISP treatment to limit the overall acute infiltration of neutrophils following SCI. Reducing neutrophil infiltration is shown to attenuate the extent of tissue damage after SCI [46, 47, 58] in which may have contributed to the functional recovery observed after targeting LAR and PTPσ in previous studies [15, 30].

The second phase of inflammation involves the recruitment of macrophages to the injury site [17, 18]. Macrophage infiltration is necessary following injury as suppressing the M2 population chronically by ablating the macrophage cellular pool is associated with worse outcomes in mice SCI [51]. The impact of resident microglia and infiltrating macrophages on the repair process is determined by their activation state, being classically activated M1, or alternatively activated M2 [17, 21]. Initially, there is a balanced ratio between pro-inflammatory M1 and pro-regenerative M2 microglia/macrophages in the SCI lesion. However, as injury progresses, this landscape switches predominantly toward an M1 phenotype forging cell death and tissue degeneration [17, 21, 59].

Our immunophenotyping studies revealed that disruption of LAR and PTPσ signaling by ILP/ISP promotes M2 macrophages as well as an overall increase in the expression of key pro-regenerative immune mediators, IL-10 and Arginase-1. Our findings are in agreement with recent studies where ChABC treatment also induced an overall anti-inflammatory response following SCI [27, 28]. The M2 inflammatory response is generally associated with improved outcomes after CNS pathology [24]. Transplantation of exogenous M2 cells promotes functional recovery following SCI indicating their ability to foster a regenerative program in the injured spinal cord [25].

### M2 microglia promote oligodendrocyte differentiation of NPCs through IL-10 release

The importance of microglia and their inflammatory phenotype in regulating endogenous cell differentiation is becoming increasingly more appreciated in CNS repair [60]. For example, increase in pro-inflammatory cytokines such as TNFα and IL-6 negatively affect hippocampal neurogenesis in the LPS-treated brain, whereas anti-inflammatory cytokines such as insulin-like growth factor-1 promote cell renewal [61, 62]. Here, we provide direct evidence that inhibition of LAR and PTPσ promotes M2 microglia that are potentially beneficial for NPC proliferation and oligodendrocyte differentiation. Interestingly, our in vitro NPC studies unraveled that IL-10 is critically important for M2-mediated increases in oligodendrogenesis but dispensable for NPC proliferation. Importantly, in primary cultures of NPCs, we previously established a direct role for CSPGs in restricting oligodendrogenesis through LAR- and PTPσ-dependent mechanisms without the presence of microglia or IL-10 [3]. Our previous ChABC studies in SCI also signified a negative role for CSPGs in restricting the survival, proliferation, and oligodendrocyte differentiation of endogenous and transplanted NPCs in rat SCI [5, 6]. Taken together, our new and previous findings suggest that CSPGs and LAR/PTPσ can influence oligodendrogenesis by direct signaling on NPCs and indirectly by modulation of M2 microglia and IL-10 expression. Similarly, Miron et al. recently demonstrated the benefits of M2 cells in promoting maturation and differentiation of OPCs in vitro and in demyelinating conditions [24]. This evidence collectively supports the importance of microglial response on endogenous oligodendrocyte replacement following CNS injury.

### Inhibition of LAR and PTPσ promotes microglia phagocytosis and mobilization

We have provided novel evidence that CSPGs may restrict the repair process by impairing the ability of microglia for phagocytosis through LAR/PTPσ-dependent mechanisms as ILP/ISP treatment was able to effectively reverse CSPG effects. Interestingly, in M1 microglia, inhibition of LAR/PTPσ also promoted phagocytosis in the absence of CSPGs suggesting that these receptors may interact with other ligands or have other functions. Nevertheless, promoting the ability of microglia for phagocytosis is beneficial for the repair process [17]. It is known that impaired phagocytosis by microglia is correlated with limited tissue regeneration in traumatic CNS injuries and neurodegenerative diseases [17, 26, 63]. Here, we provide evidence that suggests a plausible link between the long-lasting upregulation of CSPGs after SCI and the impaired clearance of debris in the injured spinal cord. Interestingly, we demonstrate that inhibiting LAR and PTPσ enhances microglia mobility on a CSPGs substrate. Migration of activated microglia to the site of spinal cord and brain injury is important for their contribution to the repair process including phagocytosis of debris and wound healing [17, 64]. In vivo imaging of microglia has identified them as highly motile cells in their environment [64, 65]. Our data suggest that the presence of CSPGs limit microglia mobility, and LAR and PTPσ appear to partially mediate this effect as ILP/ISP treatment was able to significantly attenuate the negative effects of CSPGs on microglia mobilization. Taken together, our new findings substantiate an impact for LAR and PTPσ signaling in mediating the restrictive effects of CSPGs on microglia, and demonstrate the importance of their inhibition in harnessing the potential of microglia in supporting endogenous repair processes after SCI.

### CSPGs regulate microglia response through LAR and PTPσ and a Rho/ROCK mechanism

We have unraveled that Rho activation appears to be a mechanism by which CSPGs promote a pro-inflammatory phenotype in microglia. We identified that upon exposure to CSPGs microglia elevate their Rho activity downstream to LAR and PTPσ signaling*.* Previous work by our group and others also identified that blocking the Rho/ROCK pathway overcomes CSPG effects in multiple cell types in vitro, including OPCs [29], NPCs [3], and neurons [31, 66–68]. Genetic knockout studies in primary cerebellar granule neurons revealed that both LAR and PTPσ independently mediate CSPGs ability to activate the Rho/ROCK pathway [31]. Our previous studies in spinal cord-derived NPCs also substantiated that CSPGs restrict multiple properties of NPCs by activation of the Rho/ROCK pathway [3]. We showed that CSPGs mediate their effects on NPCs through both LAR and PTPσ receptors, and therefore their co-inhibition was required to block negative effects of CSPGs. Interestingly, a ROCK inhibitor also reversed CSPGs negative effects on NPCs comparable to co-inhibition of LAR and PTPσ [3]. Notably, drugs which target the Rho/ROCK pathway have shown efficacy in promoting repair and functional recovery after SCI [67–72].

Although LAR and PTPσ receptors play an important role in CSPGs associated signaling cascade [14, 31, 73, 74], CSPGs are also shown to bind to Nogo66 receptors, NgR1 and NgR3 [75]. Nogo66 receptors were originally identified for their role in mediating the inhibitory effects of the myelin-associated inhibitor Nogo on axon regeneration in the CNS [76, 77]. However, studies by Dickendesher and colleagues identified that NgR1 and NgR3 also show high affinity binding to CSPGs [75]. Accordingly, with inhibition of LAR and PTPσ with ILP/ISP, CSPGs may still exert their effects by signaling through other mechanisms including NgR1 and NgR3. Moreover, PTPσ and LAR have shown the affinity to interact with other ligands such as heparan sulfate proteoglycan (HSPGs) [1, 78, 79]. Interestingly, interaction between HSPGs-PTPσ and CSPGs-PTPσ has different outcomes. HSPGs-PTPσ activation promotes axon growth and synapse formation [73, 79], whereas CSPG-PTPσ signaling inhibits regeneration [14, 73]. Similarly, while LAR appears to bind to CSPGs with high affinity [15], it also interacts with HSPGs [78] and homophillically to itself [80, 81]. Thus, ILP/ISP therapy in our studies may have influenced interaction of LAR and PTPσ with other ligands.

## Conclusion

In conclusion, we provide novel evidence that CSPGs promote a predominantly pro-inflammatory microenvironment in the injured spinal cord. LAR and PTPσ receptors mediate immunomodulatory effects of CSPGs, and their inhibition activates a supportive and regulatory immune response in SCI. Intracellularly, we show that LAR and PTPσ signaling appears to regulate microglia by activating the RhoA/ROCK pathway. Thus, our work has uncovered a key role for LAR and PTPσ in neuroinflammation after SCI, and provides novel insights into the mechanisms by which targeting CSPGs can ameliorate the untoward outcomes of SCI. Importantly, this work demonstrates the promise of ILP/ISP as a potential immunotherapy strategy for the treatment of SCI and other neuroinflammatory conditions of the central nervous system.

## Additional files

Additional file 1: **Figure S1.** Verification of antibody specificity for phenotypical analysis of macrophages in SCI tissues. (A-C) Flow cytometric verification of antibody specificity was performed on SCI tissue. Isolated spinal cord immune cells were stained and gated for the detection of macrophages and their pro-inflammatory (M1, CD45^+^CD68^+^CD86^+^) and pro-regenerative (M2, CD45^+^CD68^+^CD163^+^) sub-populations. Results were compared with unstained and isotype control cells for each antibody analyzed using the same gating strategy. (B) A negligible number of macrophages and their subtypes were detected in the injured isotype control compared to our injured stained group confirming the specificity of antibodies used in our macrophage panel. (C) Similarly, no significant detection was observed in unstained samples analyzed with the same gating strategies. (TIFF 20990 kb)

Additional file 2: **Figure S2.** Verification of antibody specificity for phenotypical analysis of helper T cells in SCI tissues. (A-C) Flow cytometric verification of antibody specificity for T cell detection. Isolated spinal cord immune cells were stained and gated for the detection of helper T cells and their effector (T_eff_, CD3^+^CD4^+^IFNƔ^+^) and regulatory (T_reg_, CD3^+^CD4^+^IL-10^+^) sub-populations. Results were compared with unstained and isotype control cells for each antibody analyzed using the same gating strategy. (B) A negligible number of T helper cells and their subtypes were detected in the injured isotype control compared to our injured stained group confirming the specificity of antibodies used in our T cell panel. (C) Similarly, no significant detection was observed in unstained samples analyzed with the same gating strategy. (TIFF 21021 kb)

Additional file 3: **Figure S3.** Summary of flow cytometry gating strategy used for phenotypical analysis of macrophages in injured spinal cord tissue. (A-B) For flow cytometric analysis of infiltrating macrophages, a total of 200,000 events were captured. Singlets were separated using FSC-H versus FSC-A, and CD45^+^/CD68+ cells were identified for subsequent phenotypical analysis. To identify different phenotypes of macrophages, cells were gated for CD86 as M1 or CD163 as M2 macrophages as shown in A-B. (TIFF 21098 kb)

Additional file 4: **Figure S4.** Summary of flow cytometry gating strategy used for phenotypical analysis of helper T cells in injured spinal cord tissue. (A-B) Flow cytometric analysis of infiltrating T cells started with identifying singlets using FSC-H versus FSC-A, and CD45^+^/CD3^+^/CD4^+^ cells as helper T cells. Next, to identify regulatory versus effector T cell phenotypes, cells were gated for CD4/IL-10 as T_reg_ or CD4/IFNγ as effector T cells. (TIFF 21488 kb)

Additional file 5: **Figure S5.** Microglia express LAR and PTPσ. (A-F) Immunocytochemistry on cultures of primary microglia confirms expression of LAR (A-C) and PTPσ (D-F) in microglia marked by CD11b. (TIFF 21779 kb)

## Abbreviations

aCSF: Artificial cerebrospinal fluid
BrdU: Bromodeoxyuridine
BSA: Bovine serum albumin
CNS: Central nervous system
ChABC: Chondroitinase ABC
CSPGs: Chondroitin sulfate proteoglycans
ECM: Extracellular matrix
EGF: Epidermal growth factor
FGF2: Fibroblast growth factor-2
GFAP: Glial fibrillary acidic protein
IL-10: Interleukin-10
IL-1β: Interleukin 1 beta
ILP: Intracellular LAR peptide
IMP: Intracellular Mu peptide
IFNγ: Interferon gamma
ISP: Intracellular sigma peptide
LAR: Leukocyte common antigen-related
MCM: Microglia-conditioned media
MMP: Matrix metalloproteinases
MPO: Myeloperoxidase
NPCs: Neural precursor cells
OPCs: Oligodendrocyte precursor cells
PDL: Poly-d-lysine
PTPσ: Protein tyrosine phosphatase-sigma
ROS: Reactive oxygen species
SCI: Spinal cord injury
SFM: Serum-free media
TAT: Transactivator of transcription of human immunodeficiency
TNFα: Tumor necrosis factor-α
TTBS: Tween Tris-buffered solution
