# Supplementary figures and images for: Perturbing chondroitin sulfate proteoglycan signaling through LAR and PTPσ receptors promotes a beneficial inflammatory response following spinal cord injury
Source: J Neuroinflammation. 2018 Mar 20;15:90. doi: 10.1186/s12974-018-1128-2 (PMC5861616; doi:10.1186/s12974-018-1128-2)

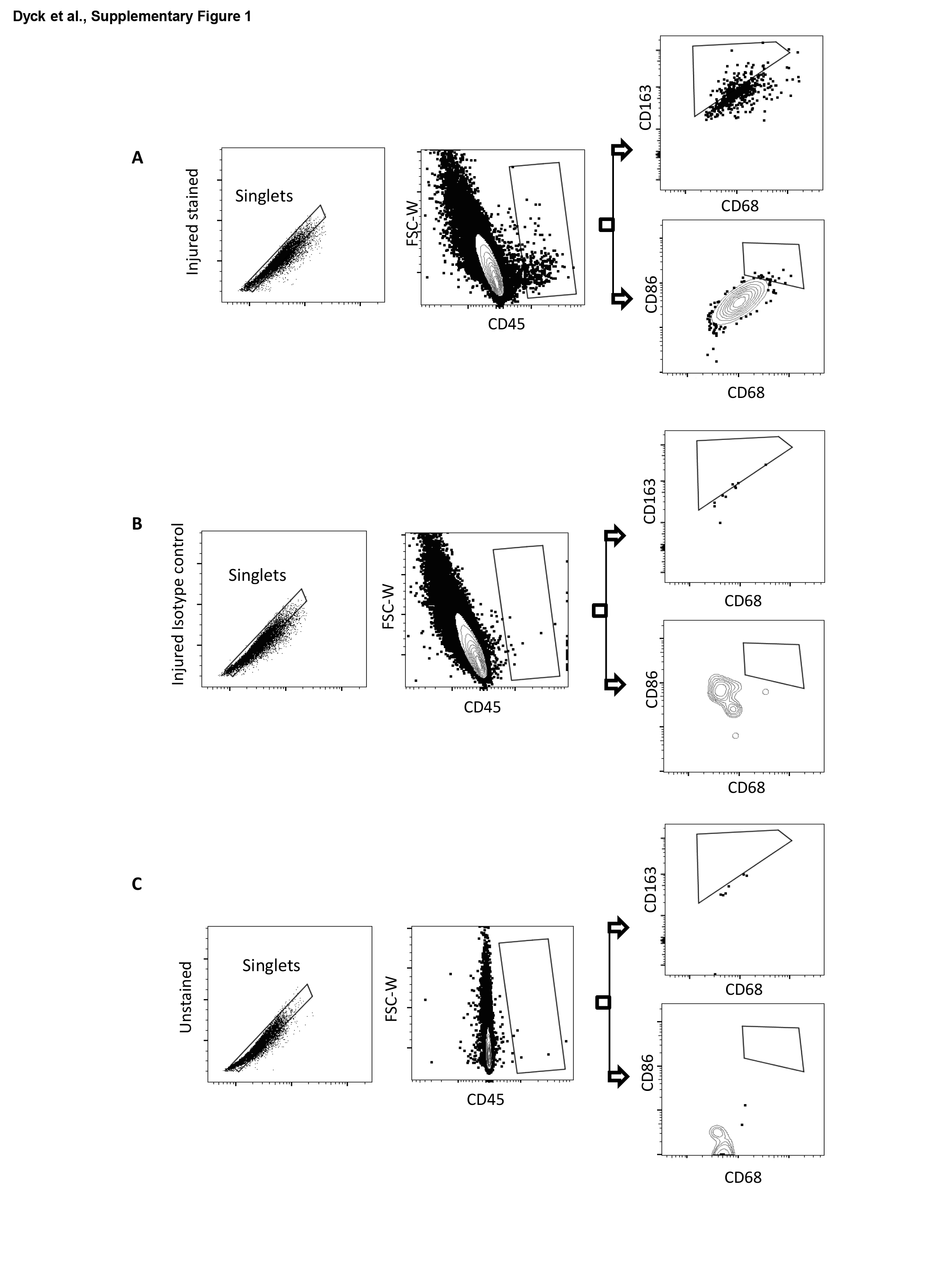

Supplement: Supplementary file 1 — Figure S1. Verification of antibody specificity for phenotypical analysis of macrophages in SCI tissues. (A-C) Flow cytometric verification of antibody specificity was performed on SCI tissue. Isolated spinal cord immune cells were stained and gated for the detection of macrophages and their pro-inflammatory (M1, CD45+CD68+CD86+) and pro-regenerative (M2, CD45+CD68+CD163+) sub-populations. Results were compared with unstained and isotype control cells for each antibody analyzed using the same gating strategy. (B) A negligible number of macrophages and their subtypes were detected in the injured isotype control compared to our injured stained group confirming the specificity of antibodies used in our macrophage panel. (C) Similarly, no significant detection was observed in unstained samples analyzed with the same gating strategies. (TIFF 20990 kb) [file 12974_2018_1128_MOESM1_ESM.tif]

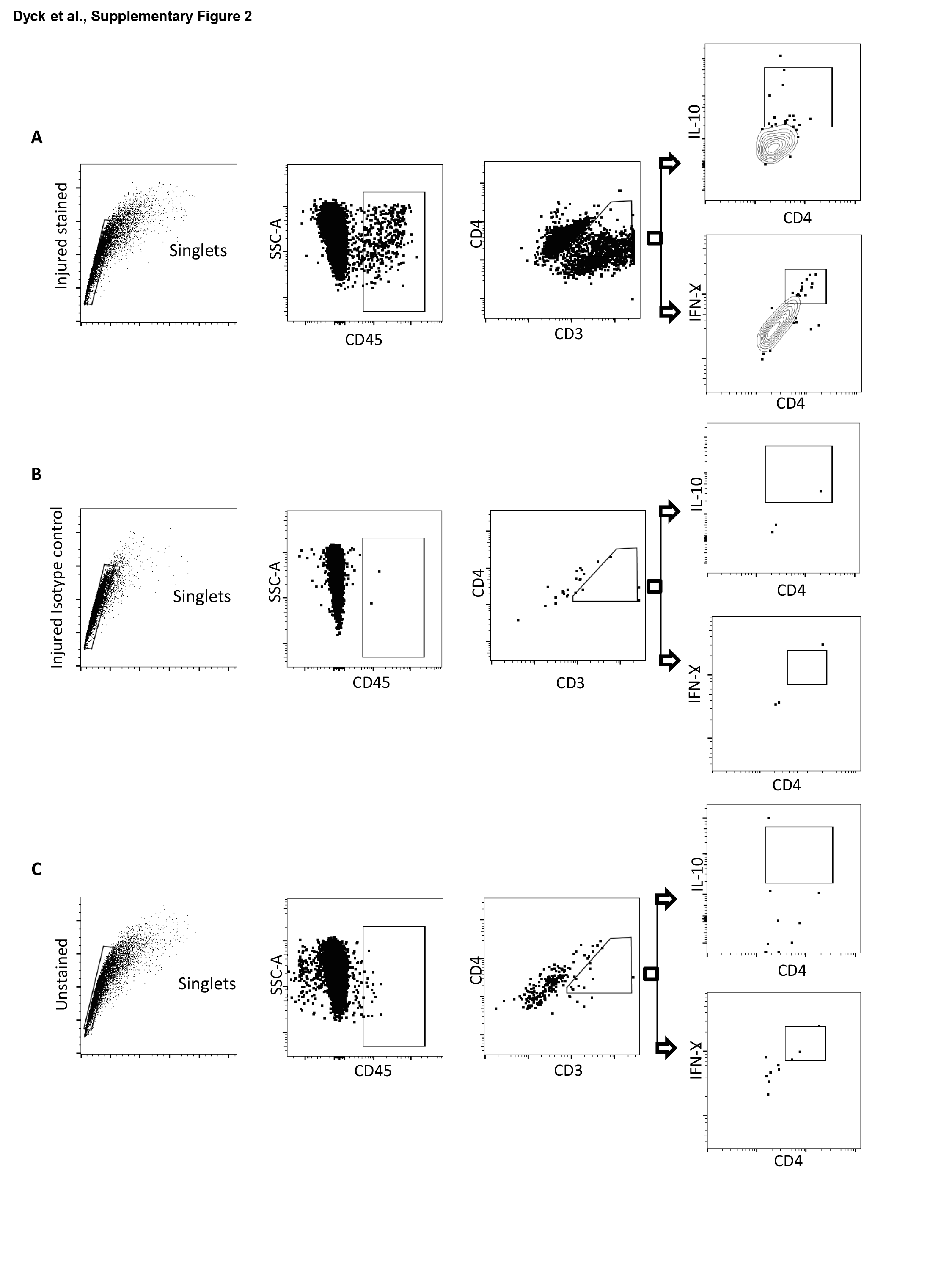

Supplement: Supplementary file 2 — Figure S2. Verification of antibody specificity for phenotypical analysis of helper T cells in SCI tissues. (A-C) Flow cytometric verification of antibody specificity for T cell detection. Isolated spinal cord immune cells were stained and gated for the detection of helper T cells and their effector (Teff, CD3+CD4+IFNƔ+) and regulatory (Treg, CD3+CD4+IL-10+) sub-populations. Results were compared with unstained and isotype control cells for each antibody analyzed using the same gating strategy. (B) A negligible number of T helper cells and their subtypes were detected in the injured isotype control compared to our injured stained group confirming the specificity of antibodies used in our T cell panel. (C) Similarly, no significant detection was observed in unstained samples analyzed with the same gating strategy. (TIFF 21021 kb) [file 12974_2018_1128_MOESM2_ESM.tif]

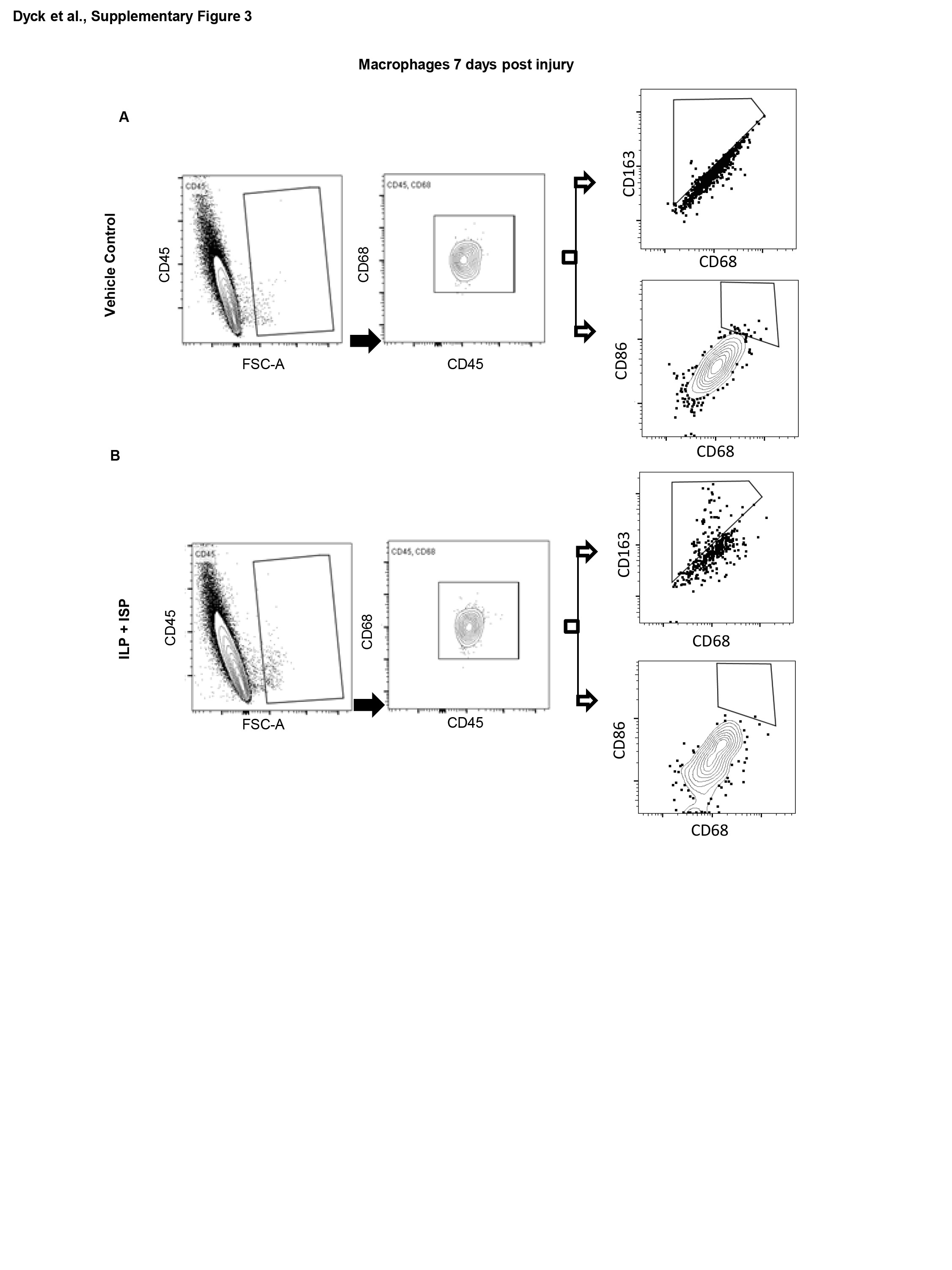

Supplement: Supplementary file 3 — Figure S3. Summary of flow cytometry gating strategy used for phenotypical analysis of macrophages in injured spinal cord tissue. (A-B) For flow cytometric analysis of infiltrating macrophages, a total of 200,000 events were captured. Singlets were separated using FSC-H versus FSC-A, and CD45+/CD68+ cells were identified for subsequent phenotypical analysis. To identify different phenotypes of macrophages, cells were gated for CD86 as M1 or CD163 as M2 macrophages as shown in A-B. (TIFF 21098 kb) [file 12974_2018_1128_MOESM3_ESM.tif]

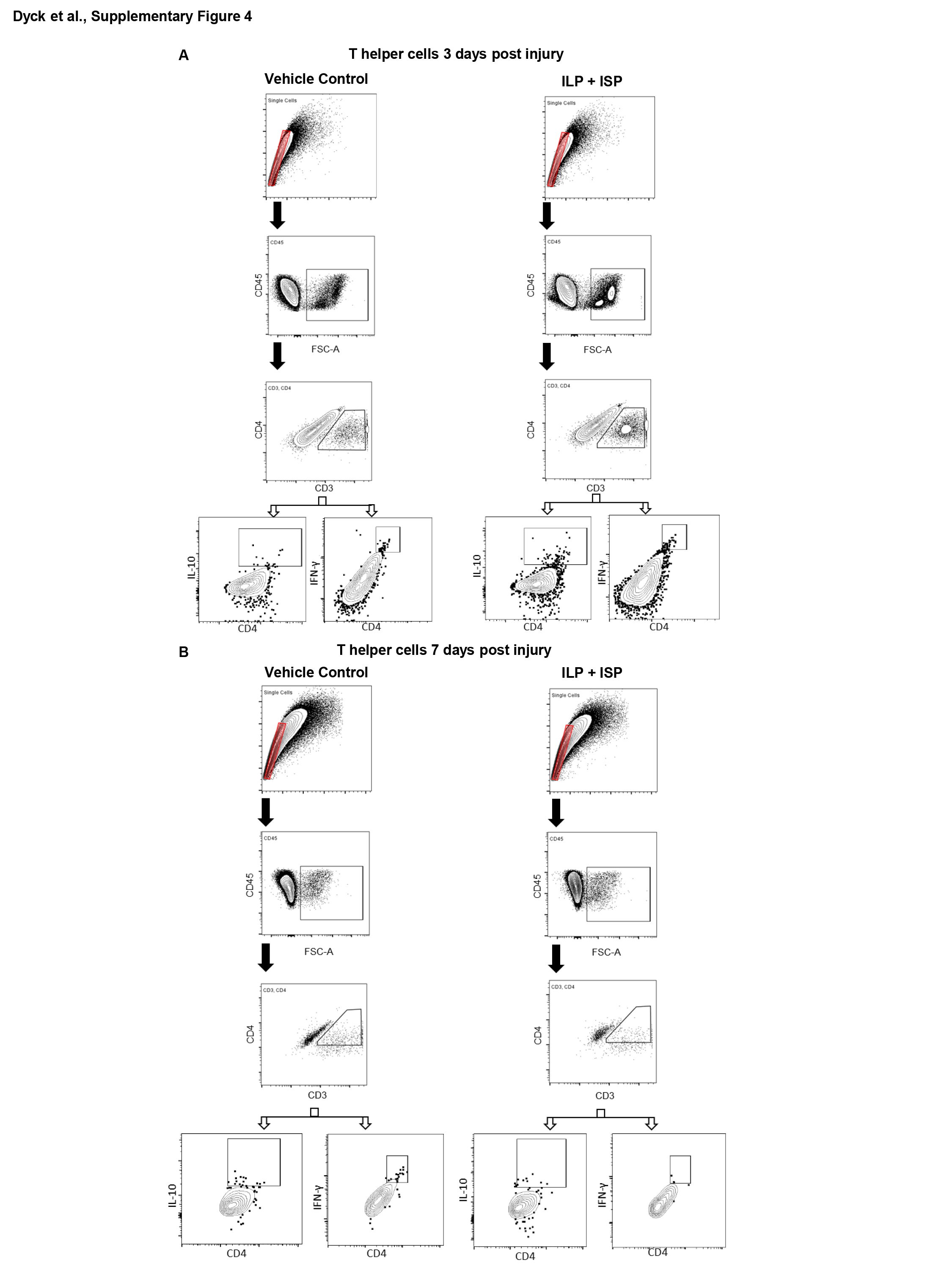

Supplement: Supplementary file 4 — Figure S4. Summary of flow cytometry gating strategy used for phenotypical analysis of helper T cells in injured spinal cord tissue. (A-B) Flow cytometric analysis of infiltrating T cells started with identifying singlets using FSC-H versus FSC-A, and CD45+/CD3+/CD4+ cells as helper T cells. Next, to identify regulatory versus effector T cell phenotypes, cells were gated for CD4/IL-10 as Treg or CD4/IFNγ as effector T cells. (TIFF 21488 kb) [file 12974_2018_1128_MOESM4_ESM.tif]

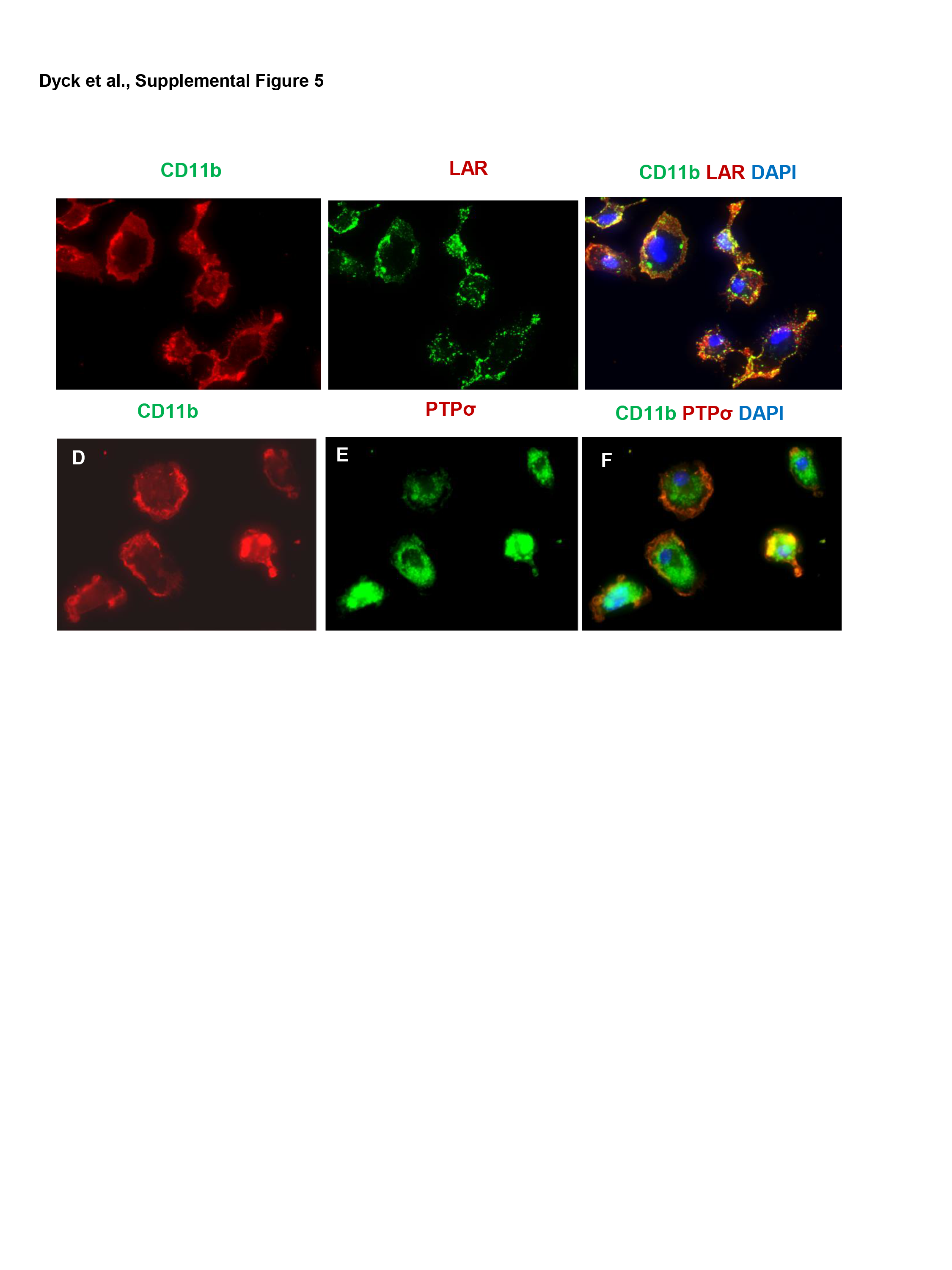

Supplement: Supplementary file 5 — Figure S5. Microglia express LAR and PTPσ. (A-F) Immunocytochemistry on cultures of primary microglia confirms expression of LAR (A-C) and PTPσ (D-F) in microglia marked by CD11b. (TIFF 21779 kb) [file 12974_2018_1128_MOESM5_ESM.tif]
